# Mutational burdens and evolutionary ages of thyroid follicular adenoma are comparable to those of follicular carcinoma

**DOI:** 10.18632/oncotarget.11922

**Published:** 2016-09-09

**Authors:** Seung-Hyun Jung, Min Sung Kim, Chan Kwon Jung, Hyun-Chun Park, So Youn Kim, Jieying Liu, Ja-Seong Bae, Sung Hak Lee, Tae-Min Kim, Sug Hyung Lee, Yeun-Jun Chung

**Affiliations:** ^1^ Department of Microbiology, College of Medicine, The Catholic University of Korea, Seoul, Korea; ^2^ Department of Pathology, College of Medicine, The Catholic University of Korea, Seoul, Korea; ^3^ Department of Integrated Research Center for Genome Polymorphism, College of Medicine, The Catholic University of Korea, Seoul, Korea; ^4^ Department of Hospital Pathology, College of Medicine, The Catholic University of Korea, Seoul, Korea; ^5^ Department of General Surgery, College of Medicine, The Catholic University of Korea, Seoul, Korea; ^6^ Department of Medical Informatics, College of Medicine, The Catholic University of Korea, Seoul, Korea

**Keywords:** follicular thyroid adenoma, follicular thyroid carcinoma, mutations, copy number alteration, tumor progression

## Abstract

Follicular thyroid adenoma (FTA) precedes follicular thyroid carcinoma (FTC) by definition with a favorable prognosis compared to FTC. However, the genetic mechanism of FTA to FTC progression remains unknown. For this, it is required to disclose FTA and FTC genomes in mutational and evolutionary perspectives. We performed whole-exome sequencing and copy number profiling of 14 FTAs and 13 FTCs, which exhibited previously-known gene mutations (*NRAS, HRAS, BRAF, TSHR* and *EIF1AX*) and copy number alterations (CNAs) (22q loss and 1q gain) in follicular tumors. In addition, we found eleven potential cancer-related genes with mutations (*EZH1, SPOP, NF1, TCF12, IGF2BP3, KMT2C, CNOT1, BRIP1, KDM5C, STAG2* and *MAP4K3*) that have not been reported in thyroid follicular tumors. Of note, FTA genomes showed comparable levels of mutations to FTC in terms of the number, sequence composition and functional consequences (potential driver mutations) of mutations. Analyses of evolutionary ages using somatic mutations as molecular clocks further identified that FTA genomes were as old as FTC genomes. Whole-transcriptome sequencing did not find any gene fusions with potential significance. Our data indicate that FTA genomes may be as old as FTC genomes, thus suggesting that follicular thyroid tumor genomes during the transition from FTA to FTC may stand stable at genomic levels in contrast to the discernable changes at pathologic and clinical levels. Also, the data suggest a possibility that the mutational profiles obtained from early biopsies may be useful for the molecular diagnosis and therapeutics of follicular tumor patients.

## INTRODUCTION

Thyroid cancer is the most common endocrine malignancy worldwide. Thyroid cancers consist of papillary (75–85%), follicular (10–20%), medullary (~5%) and anaplastic carcinomas (< 5%) [1, 2]. Follicular thyroid carcinoma (FTC) has relatively worse clinical courses than papillary thyroid carcinoma (PTC) with frequent hematogenous spread and recurrence [1]. Ten to 15% of patients with FTC develop metastatic diseases, mostly involving lung, bone and liver by hematogenous spread [3]. Approximately 11–39% of patients with FTC develop recurrence of the cancers [4, 5]. Among the four types of thyroid cancers, only FTC has a benign counterpart (follicular thyroid adenoma, FTA). FTA and FTC fall within a biologic continuum and capsular invasion and/or vascular invasion status are gold standards for distinction between FTA and FTC [1, 6]. In this scenario, FTA originated from the thyroid follicle penetrates the tumor capsule and eventually progresses to FTC. Like other tumors, FTC is considered to arise from a single clone as a result of accumulation of mutations in driver genes and subsequent clonal selection of the mutant progeny with increasingly aggressive behaviors [7]. Thus, genomic comparison of FTA and FTC genomes may provide new insights regarding genetic origins of FTC and also potential genetic determinants of the progression from FTA to FTC.

In FTC, through gene-to-gene analysis, point mutations in *RAS* (*KRAS, HRAS* and *NRAS*) and *PIK3CA,* and paired box gene 8/peroxisome proliferator-activated receptor gamma (*PAX8-PPAR*γ) gene fusion have been well-documented [2]. These alterations are also found in FTA, but less common than FTC [3], further suggesting that FTA is a genetic precursor of FTC. For example, 30% to 40% of FTCs harbor *RAS* mutations, while approximately 20% of FTAs harbor them [8]. Also, *PAX8-PPAR*γ fusion transcript is found in 4% to 13% of FTA, but 30% to 60% of FTC [9]. These genetic alterations are surely drivers for follicular tumor development, but previous genome analyses in other cancers [10, 11] suggest there should be more mutations in the FTA and FTC besides them. For a comprehensive elucidation of genetic alterations in cancers, whole-exome (WES), whole-genome or whole-transcriptome sequencing (WTS) analyses would be ideal. Recently, large-scale genome analyses for PTC and anaplastic thyroid carcinoma using next-generation sequencing have been reported [12, 13]. However, to date, there have been no such genomic studies on FTC.

In this study, to further characterize FTA and FTC genomes and extend the knowledge on genetic progression from FTA to FTC, the following questions were investigated: (i) whether FTA and FTC genomes have differences in their somatic mutation, copy numbers and gene fusion profiles and (ii) whether there are any recurrent genetic alterations that may drive FTA progression to FTC.

## RESULTS

### Whole-exome sequencing of FTA and FTC genomes

**Figure 1:**
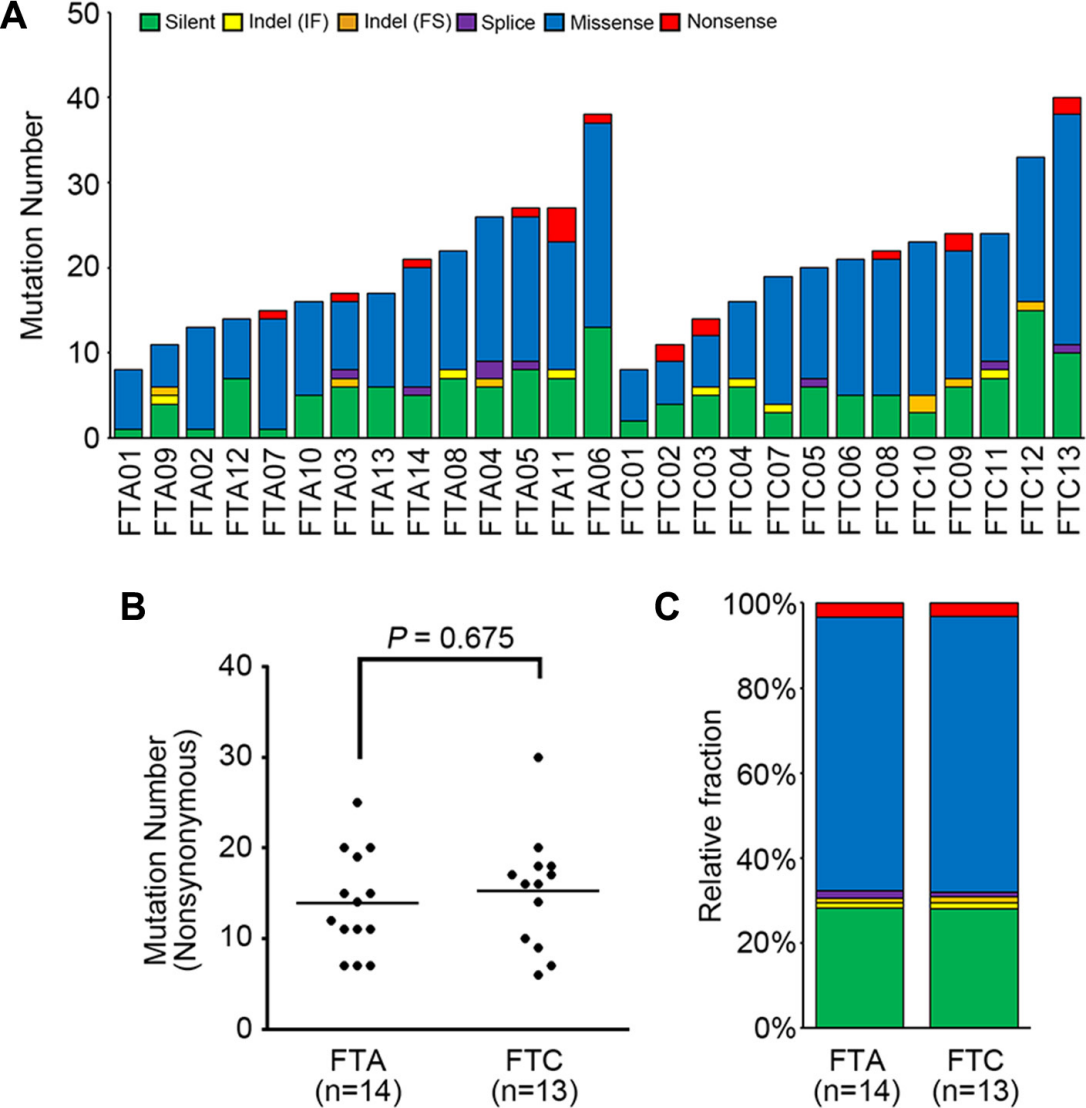
The mutational features of 27 thyroid follicular tumor genomes (**A**) The numbers of somatic mutations are shown for six functional categories. (**B**) Frequencies of nonsynonimous mutations in FTA (follicular thyroid adenoma) and FTC (follicular thyroid carcinoma) genomes. Horizontal black bars represent the mean values. No significant difference in the numbers of nonsynonymous mutations was observed between FTA (7–25; median of 12 mutations) and FTC (6–30; median of 16 mutations) genomes (*P* = 0.675). (**C**) Relative fractions of the mutations for FTA and FTC genomes are shown for the six functional categories. IF: in-frame, FS: frameshift.

To explore genomic profiles of FTA and FTC, a total of 27 thyroid follicular tumor genomes (14 FTAs and 13 FTCs) were analyzed in this study (Table 1). Coverage of depth was median of 82X (63–177X) for tumor samples and 74X (56–196X) for matched normal samples (Table S1). Using the MuTect [14] and the SomaticIndelDetector [15], we identified 8–40 point mutations and indels per sample (median of 20 somatic variants) (Figure 1A, Table S2). Two FTA patients were found to have previous history of other tumors (multiple myeloma in FTA03 and breast cancer in FTA04) (Table 1). Their mutation profiles, however, were not significantly different from other FTAs including number and sequence composition of mutations (*P* > 0.05). In the germline data of these two patients, we were not able to find any known cancer predisposition mutations. Somatic mutation density (average of 0.3 nonsynonymous mutation per Mb) of the FTCs was not significantly different with that of PTCs (*P* = 0.244) [12], but much lower than lung cancer (*P* < 0.001) or colon cancer (*P* < 0.001) [10, 16]. No significant difference in the numbers of nonsynonymous mutations was observed between FTA (7–25; median of 12 mutations) and FTC (6–30; median of 16 mutations) genomes (*P* = 0.675) (Figure 1B, Table 2). In addition, there was no significant difference between conventional and Hürthle tumor genomes (*P* = 0.327), between FTA and minimally invasive FTC genomes (*P* = 0.544), and between minimally and widely invasive FTC genomes (*P* = 0.692). Regarding the mutation spectra, no significant difference was observed between FTA and FTC genomes, either (Figure 1C). There was no correlation of the genomic features with other clinicopathologic features (Table S3).

**Table 1:** Clinocopathologic features of the patients and tumors

Case	Age/sex	Diagnosis	subtype	Size (diameter)	TNM	Extent of carcinoma	Other cancers
FTA01	48/Female	Follicular adenoma	Hürthle cell	2.0 cm	N/A	N/A	None
FTA02	35/Female	Follicular adenoma	Conventional	1.3 cm	N/A	N/A	None
FTA03	57/Male	Follicular adenoma	Hürthle cell	1.6 cm	N/A	N/A	Multiple myeloma
FTA04	71/Female	Follicular adenoma	Hürthle cell	5.4 cm	N/A	N/A	Breast cancer
FTA05	70/Female	Follicular adenoma	Conventional	3.8 cm	N/A	N/A	None
FTA06	47/Female	Follicular adenoma	Conventional	2.5 cm	N/A	N/A	None
FTA07	56/Male	Follicular adenoma	Hürthle cell	1.0 cm	N/A	N/A	None
FTA08	47/Female	Follicular adenoma	Hürthle cell	1.6 cm	N/A	N/A	None
FTA09	27/Female	Follicular adenoma	Conventional	N/A	N/A	N/A	None
FTA10	58/Female	Follicular adenoma	Conventional	1.8 cm	N/A	N/A	None
FTA11	59/Female	Follicular adenoma	Hürthle cell	1.2 cm	N/A	N/A	None
FTA12	65/Male	Follicular adenoma	Hürthle cell	0.4 cm	N/A	N/A	None
FTA13	51/Female	Follicular adenoma	Conventional	3.8 cm	N/A	N/A	None
FTA14	61/Female	Follicular adenoma	Hürthle cell	5.0 cm	N/A	N/A	None
FTC01	35/Female	Follicular carcinoma	Hürthle cell	2.2 cm	T2N1M0	Widely invasive	None
FTC02	60/Female	Follicular carcinoma	Hürthle cell	7.0 cm	T3N0M0	Minimally invasive	None
FTC03	29/Female	Follicular carcinoma	Conventional	6.0 cm	T3N0M0	Minimally invasive	None
FTC04	25/Female	Follicular carcinoma	Conventional	2.8 cm	T2N0M0	Minimally invasive	None
FTC05	39/Female	Follicular carcinoma	Conventional	3.2 cm	T2N0M0	Minimally invasive	None
FTC06	44/Female	Follicular carcinoma	Hürthle cell	4.5 cm	T3N0M0	Minimally invasive	None
FTC07	64/Male	Follicular carcinoma	Conventional	3.4 cm	T2N0M0	Minimally invasive	None
FTC08	34/Female	Follicular carcinoma	Conventional	1.9 cm	T1N0M0	Minimally invasive	None
FTC09	70/Male	Follicular carcinoma	Conventional	8.0 cm	T3N0M0	Minimally invasive	None
FTC10	73/Female	Follicular carcinoma	Conventional	N/A	N/A	Minimally invasive	None
FTC11	74/Male	Follicular carcinoma	Conventional	4.3 cm	T3N0M0	Minimally invasive	None
FTC12	59/Male	Follicular carcinoma	Hürthle cell	1.8 cm	T1N0M0	Widely invasive	None
FTC13	55/Female	Follicular carcinoma	Conventional	5.2 cm	T3N0M0	Minimally invasive	None

FTA: follicular thyroid adenoma, FTC: follicular thyroid carcinoma, N/A: not available.

**Table 2:** Summary of comparison data between FTA and FTC genomes

	FTA vs. FTC
Somatic mutation number	No significant difference
Mutation allele frequency	FTC > FTA (*P* < 0.001)
Inferred evolutionary age	No significant difference
Driver mutation number	No significant difference
Number of CNA	FTC > FTA (*P* = 0.004)

FTA: follicular thyroid adenoma, FTC: follicular thyroid carcinoma, CNA: copy number alteration.

As for variant allele frequencies (VAFs) of the point mutations, mutations of the FTC had significantly higher allele frequencies than those of the FTA (mean VAF 0.25 in FTCs and mean VAF 0.20 in FTAs, *P* < 0.001). Based on these VAFs, we inferred evolutionary ages of the 27 follicular thyroid tumor genomes. We adopted an evolutionary model that used somatic mutations as molecular clocks. In this model, the relative timing between the birth of a founder cell and the emergence of the last common ancestor before the last cycle of clonal amplification was estimated. The numbers of clonal mutations in the FTA genomes were 5 to 27 that gave conservative estimates of evolutionary ages of 200 to 1080 cell cycles. The FTC genomes showed 5 to 23 clonal mutations corresponding to 200 to 920 cell cycles of evolutionary ages. The evolutionary ages estimated from the FTC genomes were not significantly different with those estimated from the FTA genomes (*P* = 0.085, Table 2). Details of the estimated evolutionary ages are available in Figure S1.

### Copy number alterations and their distributions in FTA and FTC genomes

**Figure 2:**
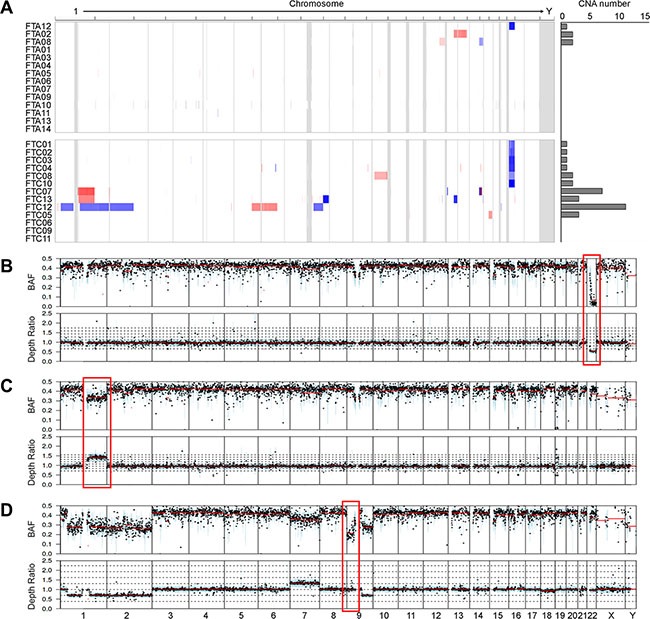
Copy number profiles and copy-neutral loss of heterozygosity (LOH) (**A**) Heatmap shows the chromosomal copy gains (red) and lesses (blue) in each sample; rows represent samples classified into FTAs (follicular thyroid adenomas) and FTCs (follicular thyroid carcinomas). Boundaries of individual chromosomes are indicated by vertical bars. On the right of heatmap, the numbers of copy number alterations (CNAs) are shown for each sample. (**B**) An example of 22q deletion in the case FTC02. The red box represents the copy number loss on chromosome 22, where *NF2* gene is located. (**C**) An example of 1q gain in the case FTC07. The red box represents the copy number gain on chromosome 1, where *NTRK1* gene is located. (**D**) The red box represents the copy-neutral LOH on chromosome 9 in the case FTC12. X-axis represnts chromosomes. BAF, B-allele frequency; Depth ratio is scaled on log_2_.

We next analyzed CNAs for the same 27 thyroid follicular tumor genomes with their matched normal genomes as references by array-CGH. A total of 37 CNAs were identified in the 13 samples (Table S4). The FTC genomes harbored significantly higher numbers of CNAs than those of the FTA genomes (mean of 0.4 (range, 0–2) for FTAs and 2.5 (range, 0–11) for FTCs, *P* = 0.004) (Figure 2A, Table 2).

Of the 37 CNAs detected, two CNA regions (gain on 1q and loss on 22q) were identified recurrently (> 2 cases) (Figure 2A). The most recurrent CNA was 22q11.1-q13.33 deletion, a 35 Mb-sized region that encompasses *NF2*, *EP300*, *MKL1* and *CHEK2* genes. Six of 13 FTCs (46%) harbored the 22q11.1-q13.33 deletion, while one of 14 FTAs (7%) harbored this deletion (*P* = 0.033) (Figure 2A). Recurrent copy number losses in chromosome 22 have been reported not only in FTCs [17, 18] but also in other human neoplasms, such as meningiomas and mesotheliomas [19]. When we examined the B allele profiles using WES data, all of 22q deletions were also shown (Figure 2B). Recurrent copy gains on 1q, where *NTRK1*, *PBX1* and *ABL2* oncogenes are located, were detected in the two FTCs (15% of FTCs) by both array-CGH and B allele analysis (Figure 2A and 2C), but none in FTAs. Recurrent copy number gains in chromosome 1q have been reported in FTCs but not in FTAs [20]. In addition to CNAs, we found that one FTC (case FTC12) harbored copy-neutral loss of heterozygosity (LOH) event on 9p24.3-p13.1 (Figure 2D), which had been reported in FTC [21].

### Driver mutations and pathways of thyroid follicular tumor genomes

**Figure 3:**
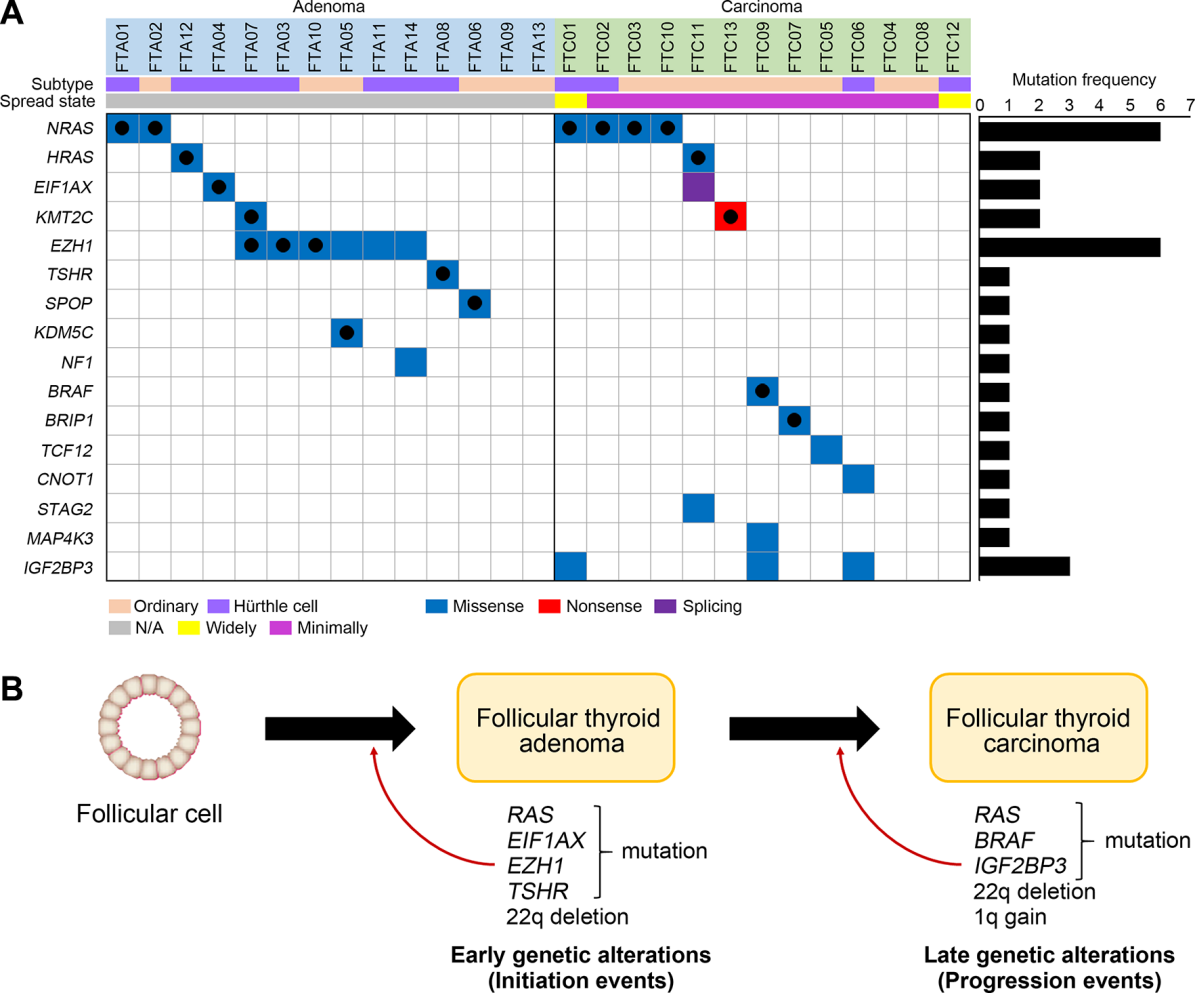
Driver mutations and pathway analyses (**A**) 16 genes with 31 nonsilent mutations are shown. On the right, the numbers of non-silent mutations are shown for each gene. Black filled circles represent the reported variants in the COSMIC database. (**B**) Schematic representation of early and late genetic alterations in thyroid follicular tumor. Development of FTA requires early genetic alteration (initiation events) such as *RAS* and *EZH1* mutations. Additional genetic alterations (progression events) such as *RAS* and *BRAF* mutations and copy loss on chromosome 22q may contribute to progression of FTA to FTC.

A total of 17 candidate driver genes with nonsilent mutations were identified based on the COSMIC (*NRAS*, *EZH1*, *HRAS*, *BRAF*, *EIF1AX*, *KDM5C*, *BRIP1*, *SPOP*, *TSHR* and *KMT2C*), recurrence (*NRAS*, *n* = 6; *EZH1*, *n* = 6; *TG*, *n* = 3; *IGF2BP3*, *n* = 3, *HRAS*, *n* = 2; *KMT2C*, *n* = 2; *EIF1AX*, *n* = 2) and the CHASM (*NRAS*, *HRAS*, *BRAF*, *NF1*, *SPOP*, *TSHR*, *TCF12*, *CNOT1*, *BRIP1*, *KDM5C*, *STAG2* and *MAP4K3*). In the CHASM analysis, mutations that significantly predicted as driver (FDR < 0.2) and overlapped either with the cancer Gene Census or the Cancer Driver Database were considered potential driver mutations. *TG* gene encodes thyroglobulin that is produced predominantly in the thyroid gland and plays essential roles for synthesis and storage of thyroid hormones [22]. Somatic *TG* mutations have been reported in autonomous thyroid adenoma and PTC [23, 24] and all *TG* mutations identified in this study were validated as somatic using Sanger sequencing (Figure S2). Functionally, however, it remains uncertain whether *TG* mutation is causally implicated in thyroid tumorigenesis. Also, *TG* is one of the largest genes in human genome, many of which can be easy targets for somatic mutation as in the case of *TTN* gene [25], suggesting that *TG* mutations are likely to be passengers. *NRAS*, *HRAS*, *EIF1AX* and *KMT2C* mutations were detected in both FTA and FTC genomes. *BRAF*, *BRIP1*, *TCF12*, *CNOT1*, *STAG2*, *MAP4K3* and *IGF2BP3* mutations were observed only in FTC while *EZH1*, *TSHR*, *SPOP*, *KDM5C* and *NF1* mutations were detected only in FTA. Overall, 16 genes with 31 nonsilent mutations were identified as potential driver mutations; *NRAS*, *HRAS*, *EIF1AX*, *KDM2C*, *EZH1*, *TSHR*, *SPOP*, *KDM5C*, *NF1*, *BRAF*, *BRIP1*, *TCF12*, *CNOT1*, *STAG2*, *MAP4K3* and *IGF2BP3* (Figure 3A, Table S2). Twelve of 14 FTAs and 10 of 13 FTCs harbored one or more potential driver mutations (Figure 3A). There was no statistical difference in the number of potential driver genes between FTAs and FTCs (*P* = 0.822, Table 2).

Next, to investigate pathway-level relationship of the individual mutations, we performed the DAVID analysis (http://david.abcc.ncifcrf.gov) and found that mutated genes in the FTCs were significantly associated with tumorigenesis-related gene functions, including ‘cell adhesion’ (*P* = 0.004), ‘MAPKinase signaling’ (*P* = 0.026) and ‘Thyroid cancer pathway (*P* = 0.048). According to the DAVID analysis, only one cancer-relate functional gene set (‘Ras protein signal transduction’, *P* = 0.011) was significantly enriched in the FTA genomes. Further details of the DAVID analysis are available in Table S5.

### Gene fusions

We explored the fusion transcripts for 10 thyroid follicular tumors (nine FTCs and one FTA) available for WTS. A total of three fusion transcripts (*MAST3-COL5A3, FAM168A-RAB6A* and *UPF3A-CDC16*) were identified from the 10 tumors (Table S6). The *MAST3-COL5A3* identified in one FTC (FTC07) is a novel fusion never reported in any database. FTC07 showed complex recombination events on chromosome 19p, where *MAST3* and *COL5A3* resides, and both genes are located at the breakpoints of the recombination, suggesting that recombination on chromosome 19p might contribute this fusion event (Figure S3). The other two fusion events (*FAM168A-RAB6A* and *UPF3A-CDC16*) have been reported in solid cancers (*FAM168A-RAB6A* in lung cancer and *UPF3A-CDC16* in thyroid cancer), both of which have the same junction breakpoints as the previous reports [26]. All three fusions were validated by reverse transcription-polymerase chain reaction (RT-PCR) followed by Sanger sequencing (Figure S4). The *PAX8-PPAR*γ, the most common fusion transcript in FTC, was not detected in our samples.

## DISCUSSION

As tumors progress, they in general become aggressive at morphologic, genetic and clinicopathologic levels. However, it remains unclear whether the clinocopathologic manifestations accompany genetic changes. Clinically, distinction between FTA and FTC is critical for proper selection of therapeutic modalities. With respect to the spatial progression of FTA to FTC, FTA is likely to precede FTC by definition. However, whether FTA progression to FTC is accompanied by genomic alterations remains unknown. To our knowledge, genome-wide mutational landscapes of neither FTA nor FTC are reported. In this study, we attempted to find whether FTA and FTC have differences in their somatic mutations and CNAs. We found that not only the quantity but also the quality of the somatic mutations was not significantly different between FTA and FTC. Only CNA numbers were significantly higher in FTC than that in FTA. Of note, FTC genomes harbored significantly higher numbers of the 22q11.1-q13.33 deletion than those of FTA genomes, suggesting this deletion would be FTC-specific.

Genomic studies on thyroid cancers have focused on PTC, the most common thyroid cancer, and to date those on FTC, the second most common, is lacking. We found that FTC genomes had similar amount of genetic alterations to the PTC genome [12] but harbored lower frequencies of somatic mutations and CNAs than other solid cancers such as lung and colon cancers that showed mutation densities around 10 per megabase [10, 16]. The earlier TCGA report on PTC and thyroid medullary carcinoma [27] genomes also showed lower frequencies of genetic alterations than those in other solid cancers [26, 28]. Only anaplastic carcinoma of thyroid, the most aggressive type, exhibited a high density of somatic mutations (~2.5 per Mb) [13]. These data suggest that thyroid cancers except for anaplastic carcinoma may need lower number of mutational events for the development than other solid cancers.

Among the 16 potential driver genes identified in the present study, *RAS* is the most recurrent gene with *NRAS* (*n* = 6) and *HRAS* (*n* = 2) identified in p.Q61K/R, the hotspot sites. The reported *RAS* mutations lies 18% to 30% in FTA and 24% to 57% in FTC [29, 30], which are in agreement with our data (21% in FTA and 38% in FTC). *EZH1* mutation was the second most recurrent mutation (*n* = 6), which had been described in 27% of FTAs [24]. Of note, all *EZH1* mutations identified in our study were found in the FTAs (43% of FTAs) along with two mutational hotspots (p.Y642F (*n* = 3) and p.Q571R (*n* = 3)), but none in FTCs. The *EZH1* p.Q571R mutation was the same variant identified in the previous study and it caused increased histone H3 trimethylation and increased proliferation of thyroid cells [24]. The other mutation p.Y642F was reported in PTC (the COSMIC database). Taken together, missense mutations of *EZH1* may play a genomic feature of FTA.

Somatic mutations of *NRAS*, *HRAS*, *BRAF*, *TSHR* and *EIF1AX* have been reported in follicular tumors while those of *EZH1*, *SPOP*, *NF1*, *TCF12*, *IGF2BP3, KMT2C, CNOT1*, *BRIP1*, *KDM5C*, *STAG2* and *MAP4K3* have not been reported in FTC (the COSMIC database). *EZH1*, *SPOP*, *NF1*, *CNOT1, BRIP1, KMT2C* and *STAG2* missense mutations have been described in PTC as well [12, 31]. By contrast, somatic mutations of *TCF12*, *KDM5C, IGF2BP3* and *MAP4K3* have not been reported in any types of thyroid cancers. At variant level, somatic mutations of *KDM5C* (p.L756I) have been identified in other cancers including lung and uterus cancers (the COSMIC database). Collectively, we confirmed that *NRAS*, *HRAS*, *EIF1AX*, *EZH1*, *TSHR* and *BRAF* mutations previously identified in follicular tumor in our FTC or FTA. Of these, *EZH1*, *TSHR* and *EIF1AX* mutations were exclusively detected in FTAs, while *BRAF* mutation was exclusively detected in FTCs. These data indicate that *EZH1*, *TSHR, EIF1AX* and *BRAF* mutations may play roles in the initial development of follicular tumors and progression of follicular tumor, respectively (Figure 3B). *RAS* mutation was detected in both FTAs and FTCs, but more common in FTCs, suggesting that it may play a role in both development and progression of follicular tumors. *TCF12*, *KDM5C, IGF2BP3* and *MAP4K3* mutations newly identified in our study might possibly be follicular tumor-specific thyroid mutations. *SPOP*, *NF1*, *BRIP*, *CNOT1, KMT2C* and *STAG2* mutations previously identified in PTC might be involved in thyroid tumorigenesis in a type-nonspecific manner. However, since these mutations in six genes are singleton mutations, further studies in a larger cohort are necessary to confirm our hypothesis.

Until now, there exist few mutation data on preneoplastic conditions or early cancers at whole-genome or whole-exome level. Of note, in breast (ductal carcinoma *in situ vs.* invasive ductal carcinoma) [32] and gastric (early *vs*. advanced gastric cancers) cancers [33], even the early primary tumors (ductal carcinoma *in situ* and early gastric cancer) are already matured in terms of the quantity and quality of the mutations. Also, microsatellite-unstable colon adenomas are known to be nearly as old as invasive colon cancers [34]. By contrast, uterine cervix and prostate cancers showed far different genetic progression pattern. For examples, carcinoma *in situ* of uterine cervix and high-grade prostate intraepithelial tumor exhibit significantly lower numbers of mutation than their corresponding invasive cancers [35, 36]. Our mutation-based evolutionary analyses revealed that the time intervals required from the initiation of a tumor to the emergence of the last common ancestor were similar between FTA and FTC genomes. The evolutionary analysis data also support our WES data that showed no significant differences between FTA and FTC genomes. Together, like breast and gastric cancers, these data indicate that FTA genomes may be as old as FTC genomes, thus suggesting that follicular thyroid tumor genomes during the transition from FTA to FTC may stand stable at genomic levels in contrast to the discernable changes at pathologic and clinical levels [6, 37]. However, because we included only two cases of widely invasive FTC, it might be better to say that the genomic difference between FTA and minimally invasive FTC might be insignificant, supporting the findings that minimally invasive FTCs can have very few pathologic differences from FTAs and act very benignly with different treatment recommendations when compared to widely invasive FTC's [38]. As for the Hürthle cell FTCs, they have some differences in biological behavior and molecular mechanisms as compared to the conventional FTC [38]. However, our study suggests that such differences are not evident in mutational profiles at least of FTA and minimally invasive FTCs.

The CNA profiles in the present study were largely consistent with those in the earlier studies [18, 39]. Unlike mutations, the FTC genomes harbored significantly higher numbers of CNAs than those of the FTA genomes. Of note, copy number loss on 22q (46% of FTCs) and copy gain on 1q (15% of FTCs) were detected recurrently in FTCs, while only one FTA (FTA12) harbored copy number loss on 22q. Earlier study showed that 22q loss and 1q gain were detected in FTAs albeit less common than FTCs [17], supporting our findings. Together, our data and the previous data indicate that 22q loss and 1q gain play roles in both development and progression of follicular tumors. In the WES data, two FTAs (case FTA09 and 13) and three FTCs (cases FTC04, 08 and 12) did not harbor any driver mutations. FTC04 and 08 harbored 22q loss and FTC12 harbored the greatest number of CNAs (Figure 2A), suggesting a possibility that CNAs might precede somatic mutations in FTCs. The FTA without any driver mutation or CNA but with several non-driver mutations could be a naïve tumor that is too young to harbor clonal driver mutations.

In the present study, we couldn't find any *PAX8-PPAR*γ fusion transcript by either WTS or RT-PCR. *PAX8* gene is essential for the genesis of the thyroid follicular cell lineage [40], and *PAX8-PPAR*γ fusion transcript is frequently detected in FTC [9, 41]. However, frequency of *PAX8-PPAR*γ fusion transcript is very low (0%~4%) in Asia [42–44], which is in agreement with our data. Rather than this, we detected three fusion transcripts (Table S6). However, all the three fusion (*MAST3-COL5A3, FAM168A-RAB6A* and *UPF3A-CDC16* fusions) were predicted to be out-of-frame and actually did not retain any intact open reading frame. Moreover, cancer-related functions in any of the 6 fusion partner genes have not been known. Our observations suggest that gene fusion transcripts besides *PAX8-PPAR*γ may not play a major and recurrent role in FTC development.

In summary, our data for the first time analyzed genomic profiles of thyroid follicular tumors and found previously unreported eleven somatic mutations in FTAs or FTCs. Our data suggest that the time course from FTA to FTC progression at genomic levels may be different from that at pathologic levels (capsular invasion and/or vascular invasion). It is theoretically possible that the uncoupling between genomic and pathologic findings could arise from non-genetic factors such as tissue microenvironment. Finally, the early genomic maturation or aging of FTA might emphasize strategies for the genomic diagnosis of follicular thyroid tumors.

## MATERIALS AND METHODS

### Thyroid follicular adenoma and carcinoma tissues

Tumor and matched normal thyroid tissues obtained by surgeries from 14 patients with FTA (six conventional and eight Hürthle cell variants) and 13 patients with FTC (nine conventional and four Hürthle cell variants) were obtained from the Tissue Banks of Seoul St. Mary Hospital of Catholic University (Seoul, Korea), Guro Hospital of Korea University (Seoul, Korea), Pusan National University Hospital (Pusan, Korea) and Keimyung University Dongsan Hospital (Daegu, Korea). All 27 patients were Korean, and none had evidence for hereditary thyroid tumors or other cancers except two cases (multiple myeloma in FTA03 and breast cancer in FTA04). Approval for this study was obtained from the institutional review board at the Catholic University of Korea, College of Medicine. Clinicopathologic features and histology of the patients are summarized in Table 1 and Figure S5, respectively. Initially, frozen tissues from the tissue banks were cut, stained with the hematoxylin/eosin and examined under microscope by a pathologist, who identified areas rich in FTA cells or FTC cells in the frozen tissues. In order to have matched normal tissues from each patient, we used thyroid tissues that were confirmed to be free of tumor cells by examination under the microscope. The purities of FTA and FTC cells were approximately 90% and 90%, respectively. For genomic DNA and RNA extraction from the frozen tissues, we used the DNeasy Blood & Tissue Kit (Qiagen, Hilden, Germany) and mirVana miRNA Isolation Kit (Invitrogen, Carlsbad, CA, USA), respectively.

### Whole-exome sequencing

WES was performed for the genomic DNA obtained from tumors and matched normal tissues using the Agilent SureSelect Human All Exome 50 Mb kit (Agilent Technologies, Santa Clara, CA) and Illumina HiSeq2000 platform according to the manufacturer's instructions. Acquisition and processing of the sequencing data were performed as previously described [33]. A Burrows-Wheeler aligner was used to align the sequencing reads onto the human reference genome (UCSC hg19). The aligned sequencing reads were evaluated using Qualimap [45], and the sequences were deposited in the SRA database (Project ID: PRJNA320003).

### Identification of somatic variants and driver mutation

Somatic variants were identified using MuTect [14] and SomaticIndelDetector [15] for point mutations and indels, respectively. The ANNOVAR package [46] was used to select somatic variants located in the exonic sequences and to predict their functional consequences. To obtain reliable and robust mutation calling, the following somatic variants were eliminated: (i) read depth fewer than 20 in either tumor or matched normal; and (ii) polymorphisms referenced in either 1000 Genomes Project or Exome Aggregation Consortium or Exome Sequencing Project with a minor allele frequency greater than 2% in Asian. Among the filtered variants, mutations overlapped with COSMIC database were manually rescued. We used the CHASM analysis with ‘thyroid’ option for the cancer tissue type in order to identify putative cancer-related mutations [47]. In addition, we looked up the cancer Gene Census [48] and Cancer Drivers Database [49]. Mutations that significantly predicted in the CHASM analysis (FDR < 0.2) and overlapped with the cancer Gene Census (http://cancer.sanger.ac.uk/census) or Cancer Driver Database (https://www.intogen.org/) were considered as potential driver mutations.

### DNA copy number and LOH analysis

DNA copy number profiling was performed using Agilent Sure Print G3 Human comparative genomic hybridization (CGH) Microarray 180K (Agilent Technologies). The genomic DNA from thyroid tumor genomes were hybridized onto the array with genomic DNA from matched normal genomes as described elsewhere [36]. Background correction and normalization form array images were done using the Agilent Feature Extraction Software v10.7.3.1. The RankSegmentation statistical algorithm in NEXUS software v7.5 (Biodiscovery Inc., El Segundo, CA) was used to define copy number alterations (CNAs) of each sample. CNAs identified by array-CGH were cross validated using WES data. For copy number analysis from the exome sequencing data, ngCGH and RankSegmentation statistical algorithm in NEXUS software v7.5 were used. We inferred the loss of heterozygosity (LOH) events using the Sequenza algorithm [50]. All of the identified CNAs and LOH events were manually curated in terms of depth ratio and B allele frequency.

### Gene set analyses

To investigate the gene ontology of individual mutations, we performed the DAVID analysis (http://david.abcc.ncifcrf.gov/) [51]. The gene ontologies including ‘biological process’, ‘cellular components’ and ‘molecular function’ category and pathways including ‘KEGG pathway’, ‘PANTHER pathway’ and ‘BIOCARTA pathway’ were sorted by significance.

### Whole-transcriptome sequencing for gene fusion analyses

The mRNA of ten thyroid tumors (one FTA and nine FTCs) was converted into cDNA library using TruSeq RNA Library Preparation Kit (Illumina). WTS was performed using an Illumina HiSeq2000 platform. Sequencing reads were mapped onto the human reference genome (GRCh37, hg19). Acquisition and processing of the sequencing data were performed as previously described [52]. Fusion transcripts were identified by searching for the discordant reads and junction spanning reads by using the pyPRADA program [53].

### Evolutionary models using somatic mutations

Estimation of evolutionary ages was performed as described elsewhere [33]. In brief, we adopted an evolutionary model for colorectal cancer genome which had 5 × 10^−10^ mutations per base pair per generation [54]. Using this, we calculated the mutation rate of *r* = 50.0 × 10^6^ × 5 × 10^−10^ ≈ 0.025 per generation or cell cycle. The number of the cell divisions required to obtain *N* mutations follows a distribution with a mean of *N*/*r*. Clonally fixed somatic mutations obtained by a founder cell since its first cell division until the emergence of the last common ancestor in a single lineage were used in this analysis.

## SUPPLEMENTARY MATERIALS








