# Supplementary material for: Mutational burdens and evolutionary ages of thyroid follicular adenoma are comparable to those of follicular carcinoma
Source: Oncotarget. 2016 Sep 9;7(43):69638–48. doi: 10.18632/oncotarget.11922 (PMC5342504; doi:10.18632/oncotarget.11922)
Supplement: Supplementary file 1 [file oncotarget-07-69638-s001.pdf]

Mutational burdens and evolutionary ages of thyroid follicular adenoma are comparable to those of follicular carcinoma

Supplementary Materials

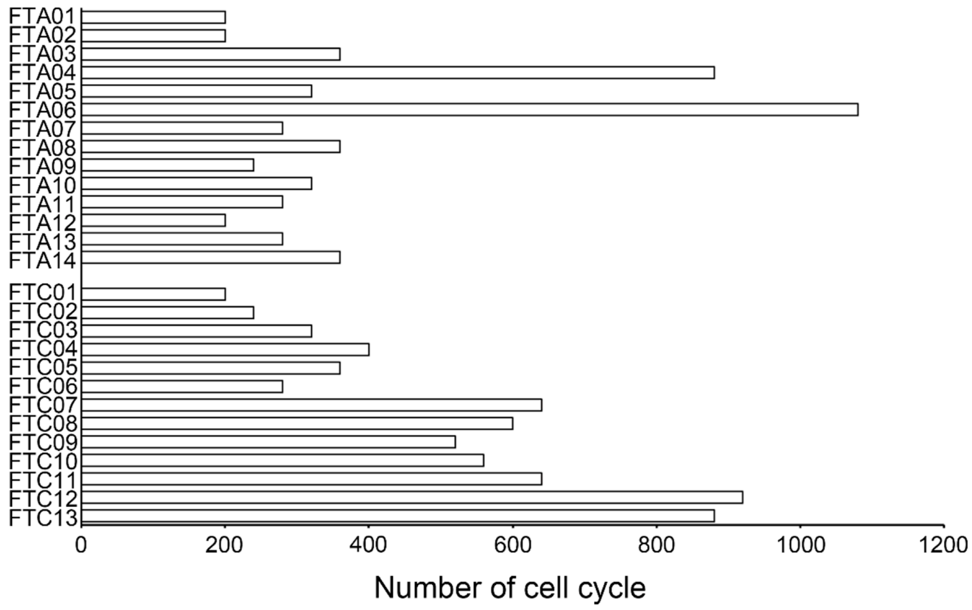

Supplementary Figure S1: Somatic mutation-based estimation of evolutionary ages. The number of cell cycles estimated by an evolutionary model that uses somatic mutations as molecular clocks are shown for 27 thyroid follicular tumor genomes.

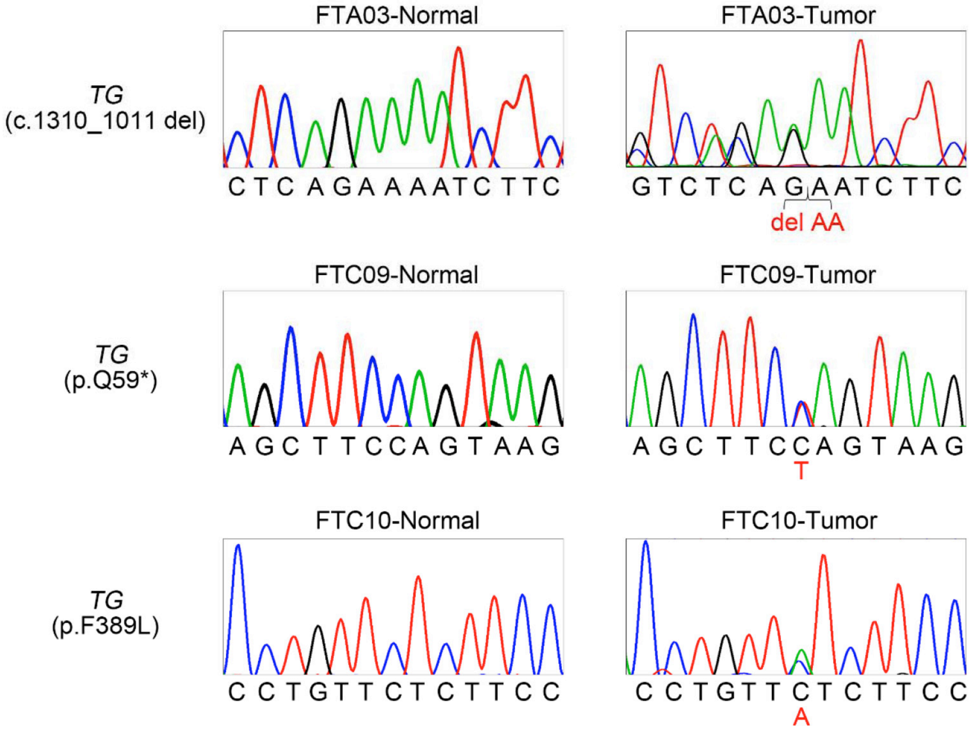

Supplementary Figure S2: Sanger sequencings of three TG gene mutations in follicular tumors with matched normal. All of the mutations were identified in tumor, but not normal and interpreted as somatic.

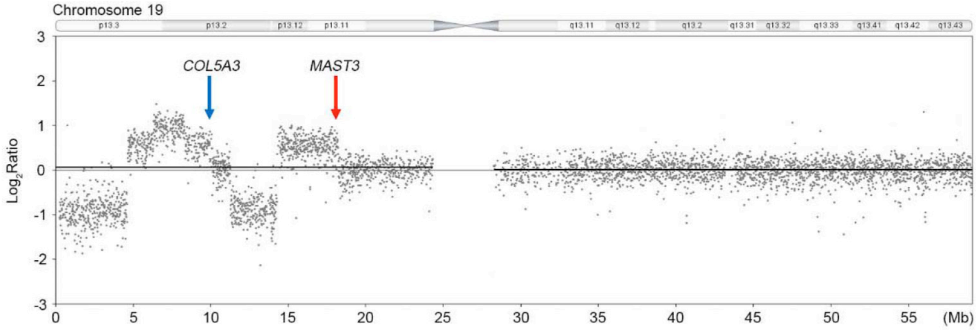

Supplementary Figure S3: The complex recombination event on chromosome 19p13.3-p13.11 in FTC07. This sample harbored *MAST3-COL5A3* fusion transcript and both genes were located on the breakpoints of copy number gains. Red and Blue arrow represent the *MAST3* and *COL5A3* gene, respectively. X-axis represents the genomic location and y-axis represents signal intensities on the $\log_2$ scale.

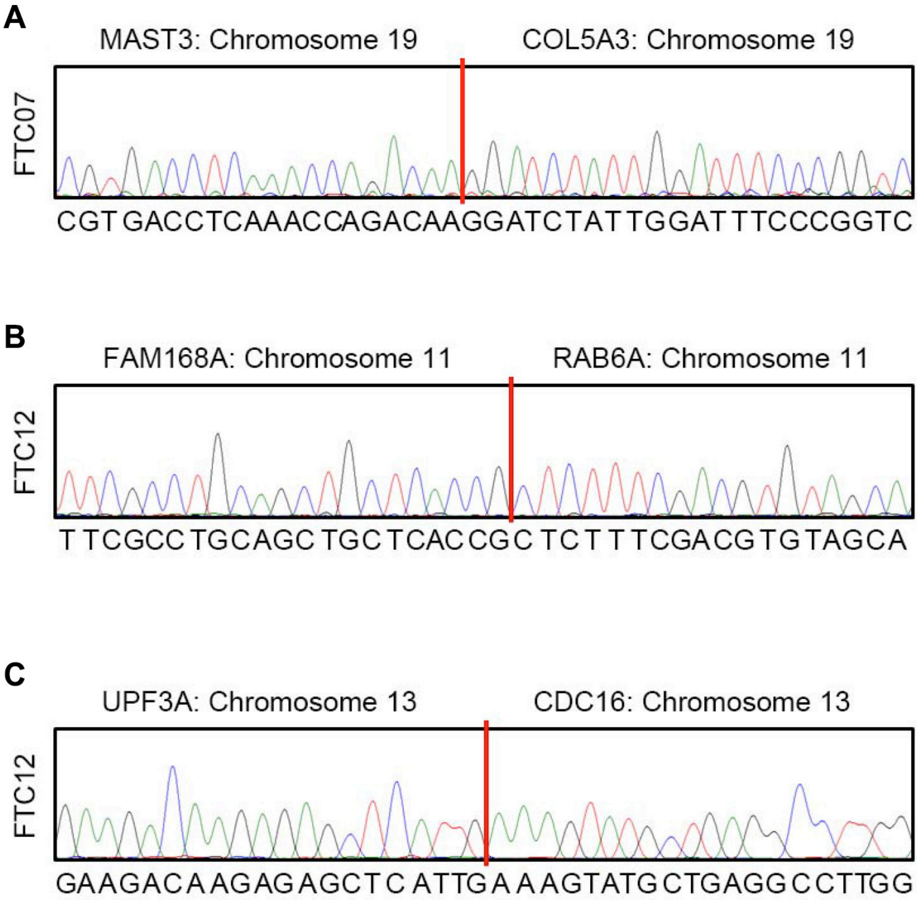

Supplementary Figure S4: Fusion transcripts identified in the thyroid follicular tumors. The *MAST3-COL5A3* identified in FTC07 is a novel fusion never reported in any database. The other two fusion events have been reported in solid cancers (*FAM168A-RAB6A* in lung cancer and *UPF3A-CDC16* in thyroid cancer); (A) *MAST3-COL5A3* (B) *FAM168A-RAB6A* (C) *UPF3A-CDC16*

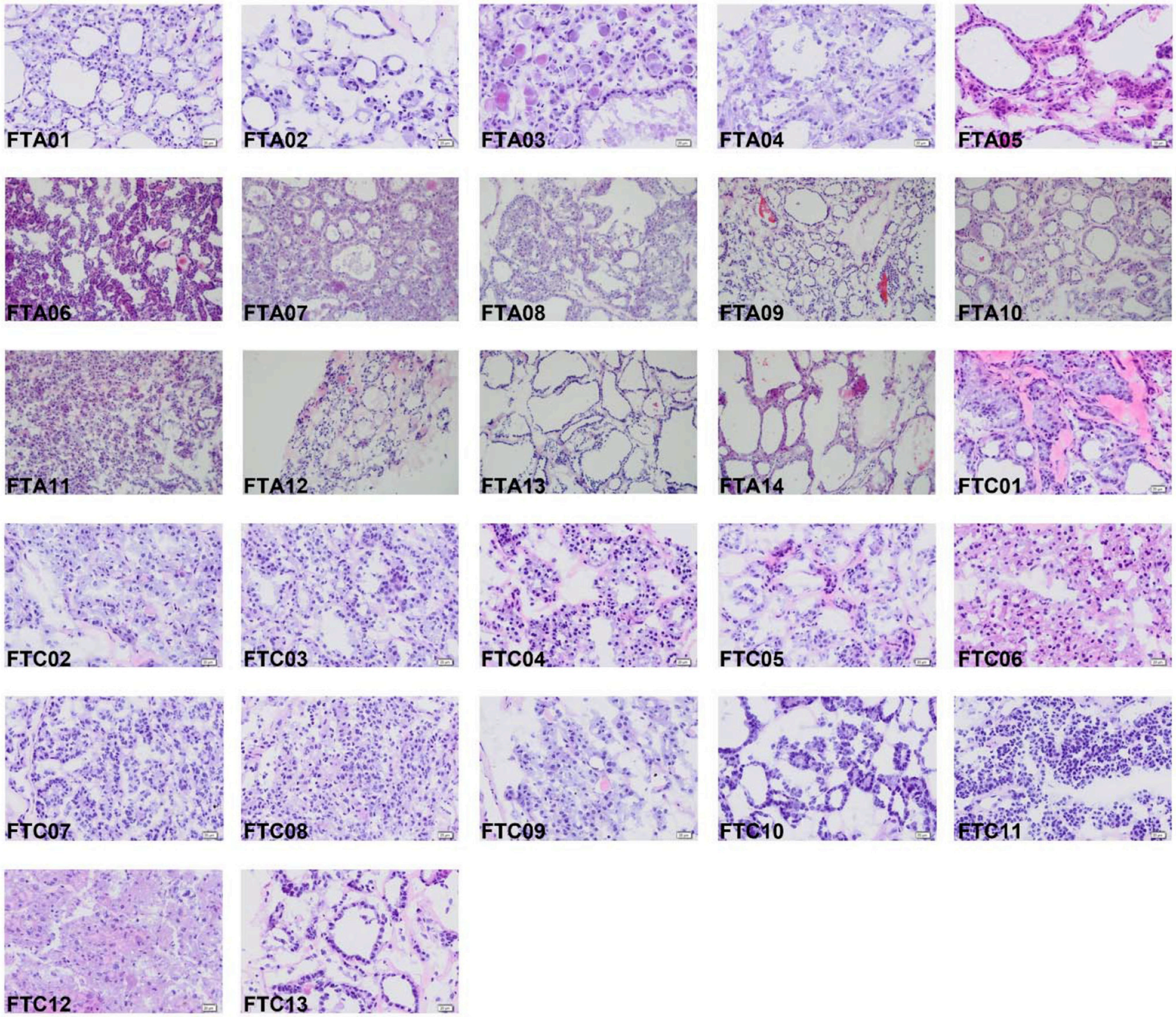

Supplementary Figure S5: Representative histology of follicular thyroid tumors. Frozen sections and hematoxylin/eosin staining of 27 follicular thyroid tumors. FTA: follicular thyroid adenoma, FTC: follicular thyroid carcinoma.

Supplementary Table S1: The description of whole-exome sequencing data

Sample ID	Sequencing reads	Mapped reads (%)	Mapped reads in exon (%)	Coverage (mean)*	Percent of bases (> = 20 reads)*
FTA01N	65,468,568	64,587,414 (98.7%)	50,268,929 (76.8%)	82.6	85.7
FTA01T	57,393,352	56,533,941 (98.5%)	44,382,347 (77.3%)	72.9	83.7
FTA02N	58,552,295	57,870,396 (98.8%)	45,095,672 (77.0%)	73.7	82.4
FTA02T	55,162,252	54,543,604 (98.9%)	43,146,859 (78.2%)	70.5	80.8
FTA03N	55,785,147	54,990,756 (98.6%)	43,278,637 (77.6%)	71.4	82.9
FTA03T	61,699,199	60,870,574 (98.7%)	48,345,376 (78.4%)	79.6	84.9
FTA04N	55,609,276	54,822,773 (98.6%)	42,883,407 (77.1%)	70.5	82.9
FTA04T	63,486,907	62,627,612 (98.7%)	49,762,055 (78.4%)	81.9	84.8
FTA05N	60,910,068	50,318,484 (82.6%)	38,498,090 (63.2%)	63.7	69.0
FTA05T	71,664,023	70,638,202 (98.6%)	54,627,819 (76.2%)	89.4	86.2
FTA06N	136,412,917	135,089,624 (99.0%)	104,244,354 (76.4%)	180.4	98.1
FTA06T	120,147,983	119,050,691 (99.1%)	89,042,151 (74.1%)	154.3	97.4
FTA07N	128,692,287	126,599,022 (98.4%)	78,311,718 (60.9%)	135.8	97.1
FTA07T	138,745,705	136,609,920 (98.5%)	88,693,507 (63.9%)	153.9	97.8
FTA08N	147,698,713	146,323,694 (99.1%)	112,456,127 (76.1%)	195.6	98.1
FTA08T	140,295,349	138,718,395 (98.9%)	101,117,655 (72.1%)	176.7	97.8
FTA09N	109,908,621	108,759,250 (99.0%)	83,110,373 (75.6%)	145.0	97.2
FTA09T	125,295,744	123,789,789 (98.8%)	87,715,579 (70.0%)	152.2	97.4
FTA10N	136,827,642	133,515,417 (97.6%)	62,424,785 (45.6%)	108.5	96.0
FTA10T	94,563,234	93,423,929 (98.8%)	73,159,957 (77.4%)	127.2	96.8
FTA11N	107,044,772	105,913,036 (98.9%)	82,015,009 (76.6%)	143.1	96.9
FTA11T	116,942,119	115,292,345 (98.6%)	79,914,857 (68.3%)	139.6	97.0
FTA12N	114,768,728	113,110,734 (98.6%)	78,362,303 (68.3%)	136.6	97.0
FTA12T	121,415,205	119,753,984 (98.6%)	80,485,430 (66.3%)	140.0	97.0
FTA13N	124,921,384	123,263,018 (98.7%)	89,992,148 (72.0%)	157.3	97.5
FTA13T	115,274,077	115,274,077 (100%)	89,222,069 (77.4%)	155.6	97.4
FTA14N	115,527,004	114,267,364 (98.9%)	85,524,036 (74.0%)	149.2	97.4
FTA14T	120,246,215	117,941,814 (98.1%)	65,293,193 (54.3%)	113.7	96.2
FTC01N	51,349,286	51,349,286 (100%)	40,776,769 (79.4%)	67.1	82.0
FTC01T	56,019,237	55,297,622 (98.7%)	43,803,045 (78.2%)	72.0	81.9
FTC02N	54,046,284	53,435,142 (98.9%)	41,295,037 (76.4%)	67.5	80.8
FTC02T	50,679,079	50,128,173 (98.9%)	39,225,031 (77.4%)	64.2	79.5
FTC03N	51,422,013	50,818,268 (98.8%)	39,197,574 (76.2%)	64.0	79.4
FTC03T	57,712,582	56,981,764 (98.7%)	43,994,783 (76.2%)	71.8	81.0
FTC04N	53,172,728	43,830,966 (82.4%)	33,486,773 (63.0%)	55.7	70.5
FTC04T	76,165,997	75,051,184 (98.5%)	57,583,363 (75.6%)	94.2	87.2
FTC05N	63,070,538	62,304,624 (98.8%)	49,001,196 (77.7%)	80.0	83.1
FTC05T	48,902,390	48,350,427 (98.9%)	38,211,637 (78.1%)	62.6	79.7
FTC06N	57,834,950	57,006,511 (98.6%)	43,715,136 (75.6%)	71.3	83.5
FTC06T	56,896,478	55,987,021 (98.4%)	42,792,897 (75.2%)	70.1	82.8
FTC07N	63,640,719	62,844,892 (98.8%)	48,660,884 (76.5%)	79.5	83.7
FTC07T	55,213,652	54,526,602 (98.8%)	42,333,159 (76.7%)	69.2	80.6
FTC08N	53,847,957	53,099,026 (98.6%)	41,160,652 (76.4%)	67.3	82.5
FTC08T	52,674,910	51,905,698 (98.5%)	40,572,639 (77.0%)	66.4	81.5
FTC09N	56,079,186	55,436,879 (98.9%)	43,186,847 (77.0%)	70.5	81.9
FTC09T	55,409,783	54,765,014 (98.8%)	43,324,795 (78.2%)	70.8	81.2
FTC10N	49,868,880	49,268,036 (98.8%)	37,669,564 (75.5%)	61.5	78.7
FTC10T	58,172,735	57,556,505 (98.9%)	44,707,174 (76.9%)	73.3	82.7
FTC11N	59,730,127	58,969,763 (98.7%)	45,514,176 (76.2%)	74.4	82.6
FTC11T	81,633,600	80,639,166 (98.8%)	62,383,638 (76.4%)	101.6	86.7
FTC12N	57,228,345	56,383,429 (98.5%)	44,619,297 (78.0%)	73.5	83.4
FTC12T	59,629,762	58,800,929 (98.6%)	46,477,988 (77.9%)	76.5	83.8
FTC13N	60,363,240	55,155,515 (91.4%)	43,928,239 (72.8%)	74.1	82.8
FTC13T	66,705,874	65,691,067 (98.5%)	50,223,254 (75.3%)	82.0	84.9

*The neoplasia and matched normal genomes are discriminated with the use of ‘T’ and ‘N’, respectively.

**The mean coverage and the % of bases (> = 20 reads) were calculated onto the targeted regions (Agilent SureSelect 50 Mb exon).

Supplementary Table S2: List of mutations identified by WES in 27 follicular tumors. See Supplementary_Table_S2

Supplementary Table S3: Correlations between genomic features and clinicopathologic features

Correlation	Correlation Coefficient	*P*-value
Age-Number of mutations	0.484	0.094
Age-Number of driver mutations	0.493	0.087
Age-Number of CNAs	–0.065	0.834
Age-Evolutionary ages	0.393	0.184
Tumor size-Number of mutations	–0.032	0.923
Tumor size-Number of driver mutations	0.557	0.060
Tumor size-Number of CNAs	–0.517	0.085
Tumor size-Evolutionary ages	–0.242	0.449

Table S4: Copy number alterations identified across 27 follicular tumors by array-CGH. See Supplementary_Table_S4

Table S5: Pathway and ontology analysis of mutations. See Supplementary_Table_S5

Supplementary Table S6: A list of fusion transcripts in 10 follicular tumors

Sample ID	Gene_A	Gene_B	A_chr (strand)	B_chr (strand)	Discordant_n	JSR_n	Junction_A	Junction_B	Evalue	A_ TAF	B_ TAF	Frame
FTC07	MAST3	COL5A3	19 (+)	19 (–)	2	5	18242857	10092794	0.11	0.2	1.0	Out-of-frame
FTC12	FAM168A	RAB6A	11 (–)	11 (–)	2	11	73308968	73390765	0.02	0.37	0.02	5UTR-CDS
FTC12	UPF3A	CDC16	13 (+)	13 (+)	3	69	115052104	115028354	2.3	0.37	0.14	Out-of-frame
