# Supplementary material for: Mutational burdens and evolutionary ages of thyroid follicular adenoma are comparable to those of follicular carcinoma
Source: Oncotarget. 2016 Sep 9;7(43):69638–48. doi: 10.18632/oncotarget.11922 (PMC5342504; doi:10.18632/oncotarget.11922)
Supplement: Supplementary file 2 [file oncotarget-07-69638-s002.docx]

**Table S2. List of mutations identified by WES in 27 follicular tumors**

| **SampleID** | **Gene** | **Genomic position^*^** | **Ref** | **Alt** | **Amino acid change** | **Exonic function** | **Cancer Drivers Database^**^** | **Cancer Gene Census^†^** | **COSMIC variant^‡^** | **CHASM^$^** |
| --- | --- | --- | --- | --- | --- | --- | --- | --- | --- | --- |
| FTA01 | NRAS | chr1:115256530 | G | T | NM_002524:c.C181A:p.Q61K | Missense | O | O | COSM580 | O |
| FTA01 | RUFY4 | chr2:218947904 | C | T | NM_198483:c.C1427T:p.A476V | Missense |  |  |  |  |
| FTA01 | COL6A3 | chr2:238285435 | T | A | NM_057166:c.A1229T:p.H410L | Missense |  |  |  |  |
| FTA01 | COL6A3 | chr2:238285436 | G | A | NM_057166:c.C1228T:p.H410Y | Missense |  |  |  |  |
| FTA01 | FOXP4 | chr6:41533594 | C | T | NM_001012426:c.C96T:p.G32G | Silent |  |  |  |  |
| FTA01 | EHMT1 | chr9:140637837 | G | T | NM_001145527:c.G838T:p.V280F | Missense |  |  |  |  |
| FTA01 | CACNA1C | chr12:2794921 | G | A | NM_001129837:c.G5617A:p.E1873K | Missense |  |  |  |  |
| FTA01 | GOLGA6B | chr15:72954595 | A | G | NM_018652:c.A850G:p.K284E | Missense |  |  |  |  |
| FTA02 | NRAS | chr1:115256529 | T | C | NM_002524:c.A182G:p.Q61R | Missense | O | O | COSM584 | O |
| FTA02 | CCDC141 | chr2:179733863 | A | G | NM_173648:c.T2375C:p.F792S | Missense |  |  |  |  |
| FTA02 | ARMC9 | chr2:232079625 | C | A | NM_001291656:c.C259A:p.R87R | Silent |  |  |  |  |
| FTA02 | ADGRV1 | chr5:89986748 | G | A | NM_032119:c.G6841A:p.A2281T | Missense |  |  |  |  |
| FTA02 | POU6F2 | chr7:39500263 | A | T | NM_001166018:c.A1520T:p.Q507L | Missense |  |  |  | O |
| FTA02 | ZNF862 | chr7:149545363 | T | C | NM_001099220:c.T781C:p.S261P | Missense |  |  |  |  |
| FTA02 | COL14A1 | chr8:121262939 | A | G | NM_021110:c.A2686G:p.N896D | Missense |  |  |  |  |
| FTA02 | NCR3LG1 | chr11:17388807 | G | A | NM_001202439:c.G673A:p.V225I | Missense |  |  |  |  |
| FTA02 | POTEM | chr14:20010235 | A | G | NM_001145442:c.T923C:p.V308A | Missense |  |  |  |  |
| FTA02 | NPIPB5 | chr16:22545785 | C | T | NM_001135865:c.C1481T:p.T494I | Missense |  |  |  | O |
| FTA02 | MYH13 | chr17:10235535 | G | A | NM_003802:c.C2179T:p.R727W | Missense |  |  |  |  |
| FTA02 | BCOR | chrX:39932750 | C | T | NM_001123383:c.G1849A:p.A617T | Missense | O | O |  |  |
| FTA02 | KIAA1210 | chrX:118222633 | T | C | NM_020721:c.A2560G:p.S854G | Missense |  |  |  |  |
| FTA03 | TP63 | chr3:189604332 | G | A | NM_001114980:c.G1217A:p.G406E | Missense |  |  |  |  |
| FTA03 | TMEM128 | chr4:4239589 | C | T | NM_001297551:c.G472A:p.V158I | Missense |  |  |  |  |
| FTA03 | LIMCH1 | chr4:41672733 | A | G |  | Splicing |  |  |  |  |
| FTA03 | BTNL3 | chr5:180432687 | C | T | NM_197975:c.C1216T:p.R406X | Nonsense |  |  |  |  |
| FTA03 | NUP205 | chr7:135333188 | C | G | NM_015135:c.C5923G:p.L1975V | Missense |  |  |  |  |
| FTA03 | TG | chr8:133898926 | GAA | G | NM_003235:c.1309_1311G | Frameshift |  |  |  |  |
| FTA03 | MUC2 | chr11:1093368 | G | A | NM_002457:c.G5187A:p.T1729T | Silent |  |  |  |  |
| FTA03 | MRGPRX1 | chr11:18955431 | C | A | NM_147199:c.G901T:p.V301L | Missense |  |  |  |  |
| FTA03 | OR5AP2 | chr11:56409476 | C | G | NM_001002925:c.G440C:p.C147S | Missense |  |  |  |  |
| FTA03 | CDK17 | chr12:96728542 | A | G | NM_001170464:c.T73C:p.L25L | Silent |  |  |  |  |
| FTA03 | MAP3K9 | chr14:71206815 | C | A | NM_001284232:c.G716T:p.S239I | Missense |  |  |  |  |
| FTA03 | ZNF267 | chr16:31927465 | A | G | NM_003414:c.A1895G:p.Y632C | Missense |  |  |  |  |
| FTA03 | EZH1 | chr17:40857116 | T | A | NM_001991:c.A1925T:p.Y642F | Missense |  |  | COSM3370737 | O |
| FTA03 | CDC27 | chr17:45216135 | G | A | NM_001293091:c.C1491T:p.D497D | Silent | O |  |  |  |
| FTA03 | CDC27 | chr17:45216150 | T | C | NM_001293091:c.A1476G:p.S492S | Silent | O |  |  |  |
| FTA03 | CDC27 | chr17:45234725 | T | C | NM_001293091:c.A318G:p.T106T | Silent | O |  |  |  |
| FTA03 | ANKRD30B | chr18:14851918 | A | G | NM_001145029:c.A3618G:p.A1206A | Silent |  |  |  |  |
| FTA04 | GON4L | chr1:155743003 | T | C |  | Splicing |  |  |  |  |
| FTA04 | LRP1B | chr2:141128340 | T | A | NM_018557:c.A10947T:p.R3649S | Missense |  |  |  |  |
| FTA04 | TTN | chr2:179396134 | T | A | NM_003319:c.A78013T:p.T26005S | Missense |  |  |  |  |
| FTA04 | CWC22 | chr2:180810292 | G | A | NM_020943:c.C2291T:p.S764L | Missense |  |  |  |  |
| FTA04 | SLC11A1 | chr2:219259446 | C | T | NM_000578:c.C1480T:p.P494S | Missense |  |  | COSM1692008 |  |
| FTA04 | SAP30 | chr4:174295178 | A | T | NM_003864:c.A530T:p.Q177L | Missense |  |  |  |  |
| FTA04 | GOLPH3 | chr5:32135679 | A | C | NM_022130:c.T471G:p.S157R | Missense |  |  |  |  |
| FTA04 | ADAM19 | chr5:156934130 | G | T | NM_033274:c.C924A:p.G308G | Silent |  |  |  |  |
| FTA04 | BTN3A3 | chr6:26452172 | G | A | NM_001242803:c.G658A:p.G220R | Missense |  |  |  |  |
| FTA04 | HIVEP2 | chr6:143081262 | AG | A | NM_006734:c.6162_6163T | Frameshift |  |  |  |  |
| FTA04 | TAF6 | chr7:99707688 | C | G | NM_001190415:c.G1278C:p.K426N | Missense |  |  |  | O |
| FTA04 | PRSS55 | chr8:10388984 | G | T | NM_001197020:c.G527T:p.C176F | Missense |  |  |  |  |
| FTA04 | CSMD3 | chr8:113349955 | G | C | NM_052900:c.C6346G:p.Q2116E | Missense |  |  |  |  |
| FTA04 | C9orf116 | chr9:138387385 | T | C | NM_001048265:c.A299G:p.N100S | Missense |  |  |  |  |
| FTA04 | RRP12 | chr10:99118341 | C | T | NM_001284337:c.G3444A:p.P1148P | Silent |  |  |  |  |
| FTA04 | ENTPD7 | chr10:101455779 | G | A | NM_020354:c.G910A:p.V304M | Missense |  |  |  |  |
| FTA04 | PKD2L1 | chr10:102058543 | C | T | NM_001253837:c.G366A:p.L122L | Silent |  |  |  |  |
| FTA04 | OR4C13 | chr11:49974485 | G | A | NM_001001955:c.G511A:p.V171I | Missense |  |  |  |  |
| FTA04 | CTAGE5 | chr14:39784004 | GTA | G |  | Splicing |  |  |  |  |
| FTA04 | PLEKHH1 | chr14:68046578 | G | T | NM_020715:c.G3168T:p.L1056L | Silent |  |  |  |  |
| FTA04 | FEM1B | chr15:68570894 | T | G | NM_015322:c.T139G:p.S47A | Missense |  |  |  |  |
| FTA04 | ELP5 | chr17:7158118 | T | C | NM_203414:c.T453C:p.C151C | Silent |  |  |  |  |
| FTA04 | OSBPL7 | chr17:45885704 | G | A | NM_145798:c.C2482T:p.R828W | Missense |  |  | COSM1260694 | O |
| FTA04 | JAK3 | chr19:17953881 | G | A | NM_000215:c.C521T:p.A174V | Missense |  | O |  |  |
| FTA04 | ASXL1 | chr20:31019128 | A | G | NM_015338:c.A723G:p.Q241Q | Silent | O | O |  |  |
| FTA04 | EIF1AX | chrX:20156731 | C | T | NM_001412:c.G26A:p.G9D | Missense | O |  |  |  |
| FTA05 | OR10R2 | chr1:158450563 | C | T | NM_001004472:c.C896T:p.T299M | Missense |  |  |  |  |
| FTA05 | SPTA1 | chr1:158626432 | C | T | NM_003126:c.G2820A:p.K940K | Silent |  |  | COSM897820 |  |
| FTA05 | FOXD4L1 | chr2:114257475 | G | A | NM_012184:c.G642A:p.L214L | Silent |  |  |  |  |
| FTA05 | TTN | chr2:179477034 | C | G | NM_003319:c.G23023C:p.E7675Q | Missense |  |  |  |  |
| FTA05 | TTN | chr2:179611319 | C | T | NM_133379:c.G15808A:p.V5270I | Missense |  |  |  |  |
| FTA05 | ACKR4 | chr3:132319910 | A | G | NM_178445:c.A669G:p.T223T | Silent |  |  |  |  |
| FTA05 | ADGRL3 | chr4:62758534 | C | T | NM_015236:c.C1437T:p.D479D | Silent |  |  |  |  |
| FTA05 | TRAM1L1 | chr4:118005694 | C | T | NM_152402:c.G856A:p.A286T | Missense |  |  |  |  |
| FTA05 | NIPBL | chr5:37002825 | C | T | NM_015384:c.C3726T:p.G1242G | Silent |  |  |  |  |
| FTA05 | LACE1 | chr6:108645124 | T | C | NM_145315:c.T235C:p.Y79H | Missense |  |  |  |  |
| FTA05 | CUL1 | chr7:148451240 | A | C | NM_003592:c.A313C:p.K105Q | Missense | O |  |  |  |
| FTA05 | CSMD1 | chr8:3216706 | C | T | NM_033225:c.G3272A:p.R1091H | Missense |  |  | COSM454470;COSM454471 | O |
| FTA05 | TRAPPC9 | chr8:141370200 | T | A | NM_001160372:c.A1444T:p.R482X | Nonsense |  |  |  |  |
| FTA05 | FOXD4 | chr9:117713 | T | C | NM_207305:c.A407G:p.D136G | Missense |  |  | COSM4163271 |  |
| FTA05 | TRPM3 | chr9:73151265 | C | A | NM_206944:c.G4239T:p.V1413V | Silent |  |  |  |  |
| FTA05 | WDR37 | chr10:1170957 | G | T | NM_014023:c.G1346T:p.S449I | Missense |  |  |  |  |
| FTA05 | AGAP5 | chr10:75457492 | A | G | NM_001144000:c.T22C:p.C8R | Missense |  |  | COSM229160 |  |
| FTA05 | KAT6B | chr10:76735962 | A | G | NM_012330:c.A1867G:p.T623A | Missense | O |  |  |  |
| FTA05 | ANGPTL5 | chr11:101777900 | C | T | NM_178127:c.G175A:p.E59K | Missense |  |  |  |  |
| FTA05 | GLB1L3 | chr11:134188642 | G | T |  | Splicing |  |  |  |  |
| FTA05 | ERP29 | chr12:112460427 | A | C | NM_006817:c.A757C:p.K253Q | Missense |  |  |  |  |
| FTA05 | ACTR10 | chr14:58701104 | A | G | NM_018477:c.A1089G:p.A363A | Silent |  |  |  |  |
| FTA05 | MPI | chr15:75189992 | T | C | NM_001289156:c.T1043C:p.I348T | Missense |  |  |  |  |
| FTA05 | EZH1 | chr17:40858152 | T | C | NM_001991:c.A1712G:p.Q571R | Missense |  |  |  |  |
| FTA05 | KDM5C | chrX:53228048 | G | T | NM_004187:c.C2266A:p.L756I | Missense | O | O | COSM756953 | O |
| FTA05 | DACH2 | chrX:85769338 | G | A | NM_001139514:c.G545A:p.R182H | Missense |  |  | COSM174097;COSM174096 |  |
| FTA05 | TCEAL3 | chrX:102864334 | G | A | NM_001006933:c.G342A:p.T114T | Silent |  |  |  |  |
| FTA06 | TMEM234 | chr1:32686759 | G | A | NM_019118:c.C208T:p.L70F | Missense |  |  |  |  |
| FTA06 | ITPKB | chr1:226829802 | C | T | NM_002221:c.G2271A:p.T757T | Silent |  |  |  |  |
| FTA06 | RYR2 | chr1:237947304 | G | A | NM_001035:c.G12292A:p.V4098I | Missense |  |  |  |  |
| FTA06 | TBC1D8 | chr2:101646060 | A | C | NM_001102426:c.T2070G:p.Y690X | Nonsense |  |  |  |  |
| FTA06 | CNTNAP5 | chr2:125530478 | G | A | NM_130773:c.G2633A:p.R878Q | Missense |  |  |  |  |
| FTA06 | SPAG16 | chr2:214204989 | C | G | NM_024532:c.C639G:p.L213L | Silent |  |  |  |  |
| FTA06 | SPCS1 | chr3:52740228 | C | T | NM_014041:c.C167T:p.P56L | Missense |  |  |  |  |
| FTA06 | GK5 | chr3:141944268 | C | T | NM_001039547:c.G30A:p.Q10Q | Silent |  |  |  |  |
| FTA06 | MUC4 | chr3:195490469 | G | C | NM_138297:c.C1227G:p.V409V | Silent |  |  |  |  |
| FTA06 | PPEF2 | chr4:76797713 | G | A | NM_006239:c.C1047T:p.P349P | Silent |  |  |  |  |
| FTA06 | IL2 | chr4:123372957 | G | A | NM_000586:c.C412T:p.L138L | Silent |  |  |  |  |
| FTA06 | KHDC3L | chr6:74072620 | C | T | NM_001017361:c.C168T:p.F56F | Silent |  |  |  |  |
| FTA06 | IGF2R | chr6:160494447 | G | A | NM_000876:c.G4893A:p.K1631K | Silent |  |  |  |  |
| FTA06 | PTPRN2 | chr7:157959683 | C | T | NM_001308267:c.G736A:p.A246T | Missense |  |  |  |  |
| FTA06 | KIFC2 | chr8:145698301 | C | T | NM_145754:c.C1985T:p.S662L | Missense |  |  |  |  |
| FTA06 | FBXO10 | chr9:37541258 | T | C | NM_012166:c.A508G:p.I170V | Missense |  |  |  |  |
| FTA06 | OR13D1 | chr9:107456906 | G | A | NM_001004484:c.G204A:p.M68I | Missense |  |  | COSM232953 |  |
| FTA06 | RALGPS1 | chr9:129815156 | C | T | NM_001190728:c.C421T:p.L141F | Missense |  |  |  |  |
| FTA06 | KCNT1 | chr9:138642022 | G | C | NM_001272003:c.G189C:p.S63S | Silent |  |  |  |  |
| FTA06 | CARS | chr11:3078584 | G | A | NM_001014437:c.C14T:p.S5F | Missense |  |  |  |  |
| FTA06 | OR5P2 | chr11:7818191 | G | A | NM_153444:c.C299T:p.A100V | Missense |  |  | COSM2151931 |  |
| FTA06 | TAF6L | chr11:62554740 | C | T | NM_006473:c.C1841T:p.S614L | Missense |  |  |  |  |
| FTA06 | NR4A1 | chr12:52448964 | T | G | NM_173157:c.T852G:p.C284W | Missense |  |  |  | O |
| FTA06 | KRT7 | chr12:52627216 | C | T | NM_005556:c.C136T:p.R46W | Missense |  |  |  |  |
| FTA06 | GLIPR1L2 | chr12:75785038 | G | A | NM_001270396:c.G142A:p.E48K | Missense |  |  | COSM695066;COSM695067 |  |
| FTA06 | RNASE10 | chr14:20978725 | C | G | NM_001012975:c.C95G:p.A32G | Missense |  |  |  |  |
| FTA06 | KIF7 | chr15:90190159 | G | A | NM_198525:c.C1690T:p.H564Y | Missense |  |  |  |  |
| FTA06 | SPATA8 | chr15:97326918 | C | A | NM_173499:c.C33A:p.D11E | Missense |  |  |  |  |
| FTA06 | SRRM2 | chr16:2812558 | C | T | NM_016333:c.C2029T:p.R677C | Missense |  |  |  |  |
| FTA06 | PRSS36 | chr16:31153951 | G | A | NM_001258290:c.C1372T:p.P458S | Missense |  |  |  |  |
| FTA06 | GLG1 | chr16:74514251 | G | A | NM_001145666:c.C1682T:p.S561F | Missense |  |  |  | O |
| FTA06 | ANKFY1 | chr17:4100745 | C | T | NM_001257999:c.G1152A:p.V384V | Silent |  |  |  |  |
| FTA06 | SPOP | chr17:47696667 | G | C | NM_001007228:c.C281G:p.P94R | Missense | O | O | COSM3370781 | O |
| FTA06 | DNAH17 | chr17:76433909 | C | T | NM_173628:c.G11847A:p.K3949K | Silent |  |  |  |  |
| FTA06 | DIDO1 | chr20:61512244 | G | A | NM_001193369:c.C5064T:p.F1688F | Silent |  |  |  |  |
| FTA06 | CCDC116 | chr22:21988703 | C | T | NM_152612:c.C465T:p.S155S | Silent |  |  |  |  |
| FTA06 | NF2 | chr22:30074272 | A | G | NM_181830:c.A1285G:p.T429A | Missense | O | O |  |  |
| FTA06 | PRR34 | chr22:46449700 | G | A | NM_018280:c.C274T:p.R92W | Missense |  |  |  |  |
| FTA07 | ADAM15 | chr1:155032757 | C | G | NM_001261464:c.C2175G:p.Y725X | Nonsense |  |  |  |  |
| FTA07 | ERICH6 | chr3:150421527 | C | T | NM_152394:c.G159A:p.E53E | Silent |  |  |  |  |
| FTA07 | BRD9 | chr5:878551 | A | G | NM_001009877:c.T1031C:p.V344A | Missense |  |  |  |  |
| FTA07 | EYS | chr6:65016948 | C | T | NM_001142800:c.G6106A:p.G2036S | Missense |  |  |  |  |
| FTA07 | SYNE1 | chr6:152462443 | T | C | NM_033071:c.A24997G:p.S8333G | Missense |  |  |  |  |
| FTA07 | KMT2C | chr7:151970951 | C | T | NM_170606:c.G851A:p.R284Q | Missense |  | O | COSM1179107 |  |
| FTA07 | PTPRN2 | chr7:157370802 | C | T | NM_001308267:c.G2413A:p.V805I | Missense |  |  |  |  |
| FTA07 | MIPOL1 | chr14:37777704 | C | T | NM_138731:c.C808T:p.R270W | Missense |  |  |  | O |
| FTA07 | NIN | chr14:51226634 | C | G | NM_016350:c.G2340C:p.E780D | Missense |  |  |  | O |
| FTA07 | PALB2 | chr16:23641718 | T | A | NM_024675:c.A1757T:p.D586V | Missense |  | O |  |  |
| FTA07 | EZH1 | chr17:40857116 | T | A | NM_001991:c.A1925T:p.Y642F | Missense |  |  | COSM3370737 | O |
| FTA07 | AMER1 | chrX:63411359 | G | A | NM_152424:c.C1808T:p.A603V | Missense |  | O |  |  |
| FTA07 | AMER1 | chrX:63411360 | C | A | NM_152424:c.G1807T:p.A603S | Missense |  | O |  |  |
| FTA07 | GRIA3 | chrX:122387312 | C | T | NM_000828:c.C427T:p.P143S | Missense |  |  |  | O |
| FTA07 | PASD1 | chrX:150842437 | C | A | NM_173493:c.C1954A:p.Q652K | Missense |  |  |  |  |
| FTA08 | COL9A2 | chr1:40782828 | G | A | NM_001852:c.C42T:p.L14L | Silent |  |  |  |  |
| FTA08 | ANKRD13C | chr1:70819732 | G | C | NM_030816:c.C360G:p.V120V | Silent |  |  |  |  |
| FTA08 | GJA5 | chr1:147231016 | C | T | NM_005266:c.G331A:p.A111T | Missense |  |  |  |  |
| FTA08 | CFHR3 | chr1:196762536 | G | A | NM_001166624:c.G703A:p.D235N | Missense |  |  | COSM1689485 |  |
| FTA08 | DNAH7 | chr2:196602728 | T | C | NM_018897:c.A11992G:p.I3998V | Missense |  |  |  |  |
| FTA08 | MAML3 | chr4:140811081 | C | CTGT | NM_018717:c.1509_1509delinsACAG | Nonframeshift |  |  |  |  |
| FTA08 | AGGF1 | chr5:76332468 | G | T | NM_018046:c.G604T:p.V202L | Missense |  |  |  |  |
| FTA08 | LRRC1 | chr6:53787545 | A | G | NM_018214:c.A1529G:p.K510R | Missense |  |  |  |  |
| FTA08 | DOCK4 | chr7:111580288 | C | A | NM_014705:c.G854T:p.G285V | Missense |  |  |  |  |
| FTA08 | ANK1 | chr8:41522374 | G | A | NM_001142445:c.C168T:p.D56D | Silent |  |  |  |  |
| FTA08 | PCMTD1 | chr8:52733227 | C | T | NM_001286782:c.G530A:p.R177H | Missense |  |  |  |  |
| FTA08 | CA1 | chr8:86240836 | G | A | NM_001291968:c.C400T:p.R134C | Missense |  |  |  | O |
| FTA08 | CKS2 | chr9:91930169 | C | T | NM_001827:c.C144T:p.V48V | Silent |  |  |  |  |
| FTA08 | PAPPA | chr9:119115934 | C | T | NM_002581:c.C4209T:p.G1403G | Silent |  |  |  |  |
| FTA08 | C10orf95 | chr10:104210247 | C | T | NM_024886:c.G741A:p.R247R | Silent |  |  |  |  |
| FTA08 | GPR26 | chr10:125426323 | G | A | NM_153442:c.G400A:p.A134T | Missense |  |  |  |  |
| FTA08 | FMNL3 | chr12:50050191 | T | C | NM_198900:c.A728G:p.K243R | Missense |  |  |  | O |
| FTA08 | TSHR | chr14:81609760 | T | C | NM_000369:c.T1358C:p.M453T | Missense |  | O | COSM26449 | O |
| FTA08 | KRT9 | chr17:39724814 | C | A | NM_000226:c.G1116T:p.Q372H | Missense |  |  |  |  |
| FTA08 | ADRA1D | chr20:4228546 | G | A | NM_000678:c.C1059T:p.V353V | Silent |  |  |  |  |
| FTA08 | ITGB2 | chr21:46321561 | T | G | NM_000211:c.A587C:p.K196T | Missense |  |  |  |  |
| FTA08 | DDX53 | chrX:23018520 | C | A | NM_182699:c.C346A:p.Q116K | Missense |  |  |  |  |
| FTA09 | MMEL1 | chr1:2537013 | C | A | NM_033467:p.G267V | Missense |  |  |  |  |
| FTA09 | PGBD5 | chr1:230493006 | TG | T | NM_001258311:c.392_393A | Frameshift |  |  |  |  |
| FTA09 | IGF2BP3 | chr7:23353231 | A | G | NM_006547:c.T1437C:p.F479F | Silent |  |  |  |  |
| FTA09 | ACTR3B | chr7:152513619 | G | A | NM_001040135:c.G486A:p.T162T | Silent |  |  |  |  |
| FTA09 | DDX58 | chr9:32472997 | G | A | NM_014314:c.C1990T:p.R664C | Missense |  |  |  |  |
| FTA09 | BEST1 | chr11:61719313 | C | T | NM_004183:c.C35T:p.A12V | Missense |  |  |  |  |
| FTA09 | OAS1 | chr12:113357311 | G | A | NM_001032409:c.G1058A:p.S353N | Missense |  |  |  |  |
| FTA09 | DCLK1 | chr13:36699965 | C | T | NM_004734:c.G310A:p.G104R | Missense |  |  |  |  |
| FTA09 | PALM | chr19:746328 | C | T | NM_001040134:c.C546T:p.P182P | Silent |  |  |  |  |
| FTA09 | CACNA1A | chr19:13318232 | G | A | NM_001127222:c.C7416T:p.L2472L | Silent |  |  |  |  |
| FTA09 | KRTAP6-3 | chr21:31964896 | CGGAGGCCTGGGCTTTGGCTAT | C | NM_181605:c.132_153C | Nonframeshift |  |  |  |  |
| FTA10 | USH2A | chr1:216019214 | C | T | NM_206933:c.G9007A:p.V3003I | Missense |  |  |  |  |
| FTA10 | PCOLCE2 | chr3:142542443 | C | T | NM_013363:c.G880A:p.V294M | Missense |  |  | COSM1219857 |  |
| FTA10 | DSPP | chr4:88536460 | C | T | NM_014208:c.C2646T:p.S882S | Silent |  |  |  |  |
| FTA10 | AADAT | chr4:170994293 | G | T | NM_016228:c.C648A:p.I216I | Silent |  |  |  |  |
| FTA10 | COL19A1 | chr6:70873258 | T | A | NM_001858:c.T2370A:p.H790Q | Missense |  |  |  |  |
| FTA10 | RRAGD | chr6:90088982 | T | C | NM_021244:c.A720G:p.Q240Q | Silent |  |  |  |  |
| FTA10 | NAALAD2 | chr11:89924742 | C | A | NM_001300930:c.C1951A:p.P651T | Missense |  |  |  | O |
| FTA10 | NOP2 | chr12:6672928 | C | A | NM_001033714:c.G528T:p.W176C | Missense |  |  |  |  |
| FTA10 | ETNK1 | chr12:22796842 | A | T | NM_001039481:c.A569T:p.Q190L | Missense |  | O |  |  |
| FTA10 | HERC2 | chr15:28514562 | T | C | NM_004667:c.A1278G:p.I426M | Missense |  |  |  |  |
| FTA10 | EZH1 | chr17:40857116 | T | A | NM_001991:c.A1925T:p.Y642F | Missense |  |  | COSM3370737 | O |
| FTA10 | MPO | chr17:56349078 | G | A | NM_000250:c.C1968T:p.R656R | Silent |  |  |  |  |
| FTA10 | STRADA | chr17:61781081 | C | T | NM_001003786:c.G1063A:p.E355K | Missense |  |  |  |  |
| FTA10 | PLPP2 | chr19:288042 | G | T | NM_003712:c.C182A:p.T61N | Missense |  |  |  |  |
| FTA10 | IL1RAPL1 | chrX:29959901 | G | A | NM_014271:c.G1191A:p.E397E | Silent |  |  |  |  |
| FTA10 | FAM47A | chrX:34149921 | G | C | NM_203408:c.C475G:p.L159V | Missense |  |  |  |  |
| FTA11 | RCC2 | chr1:17764948 | G | T | NM_001136204:c.C63A:p.A21A | Silent |  |  |  |  |
| FTA11 | NOTCH2 | chr1:120611960 | C | T | NM_001200001:c.G61A:p.A21T | Missense | O | O | COSM1600651 |  |
| FTA11 | GOLT1A | chr1:204170871 | C | T | NM_198447:c.G186A:p.R62R | Silent |  |  | COSM414684 |  |
| FTA11 | OR2L3 | chr1:248224451 | T | C | NM_001004687:c.T468C:p.C156C | Silent |  |  |  |  |
| FTA11 | NEU2 | chr2:233899696 | G | A | NM_005383:c.G1072A:p.D358N | Missense |  |  |  |  |
| FTA11 | PRSS50 | chr3:46754440 | TTGA | T | NM_013270:c.869_872A | Nonframeshift |  |  |  |  |
| FTA11 | NIPBL | chr5:37006546 | A | G | NM_015384:c.A3943G:p.I1315V | Missense |  |  |  |  |
| FTA11 | CENPH | chr5:68498754 | G | A | NM_022909:c.G444A:p.W148X | Nonsense |  |  |  |  |
| FTA11 | NEURL1B | chr5:172110451 | G | T | NM_001308177:c.G61T:p.A21S | Missense |  |  |  |  |
| FTA11 | KHDRBS2 | chr6:62604641 | G | A | NM_152688:c.C709T:p.P237S | Missense |  |  |  |  |
| FTA11 | MBOAT4 | chr8:29996250 | C | A | NM_001100916:c.G142T:p.G48X | Nonsense |  |  |  |  |
| FTA11 | LMX1B | chr9:129456029 | A | C | NM_001174146:c.A824C:p.K275T | Missense |  |  |  |  |
| FTA11 | ADARB2 | chr10:1230810 | C | T | NM_018702:c.G2034A:p.L678L | Silent |  |  |  |  |
| FTA11 | PROSER2 | chr10:11908676 | G | A | NM_153256:c.G285A:p.E95E | Silent |  |  |  |  |
| FTA11 | OR4X1 | chr11:48285907 | G | A | NM_001004726:c.G495A:p.P165P | Silent |  |  | COSM1179748 |  |
| FTA11 | OR5M11 | chr11:56310557 | C | T | NM_001005245:c.G177A:p.M59I | Missense |  |  |  |  |
| FTA11 | STYK1 | chr12:10782235 | G | A | NM_018423:c.C490T:p.R164X | Nonsense |  |  | COSM934491 |  |
| FTA11 | TCTN1 | chr12:111078270 | C | T | NM_001082537:c.C926T:p.P309L | Missense |  |  |  |  |
| FTA11 | CHST14 | chr15:40764158 | C | T | NM_130468:c.C746T:p.A249V | Missense |  |  |  |  |
| FTA11 | KRTAP16-1 | chr17:39464959 | G | A | NM_001146182:c.C547T:p.L183F | Missense |  |  |  |  |
| FTA11 | KRT13 | chr17:39659673 | G | A | NM_002274:c.C601T:p.R201C | Missense |  |  | COSM3402887 |  |
| FTA11 | EZH1 | chr17:40858152 | T | C | NM_001991:c.A1712G:p.Q571R | Missense |  |  |  |  |
| FTA11 | MC2R | chr18:13884917 | G | A | NM_000529:c.C601T:p.R201X | Nonsense |  |  |  |  |
| FTA11 | TCEB3C,TCEB3CL | chr18:44555371 | G | A | NM_001100817:c.C843T:p.N281N | Silent |  |  |  |  |
| FTA11 | CEACAM3 | chr19:42312932 | C | T | NM_001277163:c.C506T:p.A169V | Missense |  |  |  |  |
| FTA11 | MYO18B | chr22:26351203 | C | T | NM_032608:c.C6029T:p.A2010V | Missense |  |  |  |  |
| FTA11 | RNF113A | chrX:119004889 | A | T | NM_006978:c.T688A:p.W230R | Missense |  |  |  |  |
| FTA12 | CELA3B | chr1:22310235 | C | T | NM_007352:c.C411T:p.D137D | Silent |  |  |  |  |
| FTA12 | POMGNT2 | chr3:43122273 | C | G | NM_032806:c.G651C:p.R217R | Silent |  |  |  |  |
| FTA12 | PDGFRB | chr5:149513317 | G | A | NM_002609:c.C766T:p.R256W | Missense |  |  |  |  |
| FTA12 | CA9 | chr9:35674208 | T | C | NM_001216:c.T252C:p.P84P | Silent |  |  |  |  |
| FTA12 | GAD2 | chr10:26569953 | G | A | NM_000818:c.G1173A:p.T391T | Silent |  |  |  |  |
| FTA12 | HRAS | chr11:533874 | T | C | NM_001130442:c.A182G:p.Q61R | Missense | O | O | COSM499;COSM244958 | O |
| FTA12 | MUC2 | chr11:1082659 | G | A | NM_002457:c.G1908A:p.A636A | Silent |  |  |  |  |
| FTA12 | FLT1 | chr13:28964048 | A | C | NM_001159920:c.T1854G:p.T618T | Silent |  |  |  |  |
| FTA12 | THSD4 | chr15:71548979 | C | T | NM_024817:c.C940T:p.R314W | Missense |  |  |  |  |
| FTA12 | KAT8 | chr16:31141854 | C | G | NM_032188:c.C1084G:p.R362G | Missense |  |  |  |  |
| FTA12 | CES1 | chr16:55853481 | G | A | NM_001025194:c.C869T:p.T290M | Missense |  |  |  |  |
| FTA12 | KRTAP4-9 | chr17:39262247 | T | G | NM_001146041:c.T607G:p.L203V | Missense |  |  |  |  |
| FTA12 | MSN | chrX:64956699 | G | A | NM_002444:c.G1002A:p.E334E | Silent |  |  | COSM403463 |  |
| FTA12 | MSN | chrX:64956743 | A | G | NM_002444:c.A1046G:p.E349G | Missense |  |  | COSM227664 |  |
| FTA13 | MYCN | chr2:16086210 | G | A | NM_001293231:c.G753A:p.R251R | Silent | O |  |  |  |
| FTA13 | RDH14 | chr2:18741730 | C | T | NM_020905:c.G109A:p.D37N | Missense |  |  |  |  |
| FTA13 | BRD9 | chr5:878551 | A | G | NM_001009877:c.T1031C:p.V344A | Missense |  |  |  |  |
| FTA13 | PRUNE2 | chr9:79320991 | C | T | NM_001308047:c.G6199A:p.A2067T | Missense |  |  |  |  |
| FTA13 | GNAQ | chr9:80646047 | G | A | NM_002072:c.C105T:p.D35D | Silent |  | O | COSM3670056 |  |
| FTA13 | GOLGA7B | chr10:99623747 | G | A | NM_001010917:c.G199A:p.E67K | Missense |  |  |  |  |
| FTA13 | SLC6A5 | chr11:20668384 | G | A | NM_004211:c.G1974A:p.L658L | Silent |  |  |  |  |
| FTA13 | RTN3 | chr11:63486412 | T | A | NM_201428:c.T381A:p.S127S | Silent |  |  |  |  |
| FTA13 | LRRC32 | chr11:76372401 | T | C | NM_001128922:c.A236G:p.D79G | Missense |  |  |  |  |
| FTA13 | MYO1A | chr12:57436937 | C | G | NM_005379:c.G1017C:p.Q339H | Missense |  |  |  |  |
| FTA13 | PIWIL1 | chr12:130845868 | G | C | NM_001190971:c.G1809C:p.K603N | Missense |  |  |  |  |
| FTA13 | MRPL57 | chr13:21751103 | G | T | NM_024026:c.G48T:p.Q16H | Missense |  |  |  |  |
| FTA13 | CSNK1D | chr17:80210988 | G | A | NM_001893:c.C469T:p.R157W | Missense |  |  |  | O |
| FTA13 | FUT5 | chr19:5867620 | G | A | NM_002034:c.C117T:p.D39D | Silent |  |  |  |  |
| FTA13 | NLRP2 | chr19:55494885 | G | A | NM_001174082:c.G1753A:p.G585S | Missense |  |  |  |  |
| FTA13 | NLRP5 | chr19:56539737 | C | T | NM_153447:c.C2138T:p.P713L | Missense |  |  | COSM3404670 |  |
| FTA13 | TRO | chrX:54956013 | A | C | NM_001271183:c.A1449C:p.T483T | Silent |  |  |  |  |
| FTC14 | PRRC2C | chr1:171519307 | A | C | NM_015172:c.A5049C:p.E1683D | Missense |  |  |  |  |
| FTC14 | CRB1 | chr1:197316487 | C | T | NM_001257966:c.C866T:p.T289M | Missense |  |  | COSM115932 |  |
| FTC14 | TTN | chr2:179477204 | A | T | NM_003319:c.T22853A:p.I7618N | Missense |  |  |  |  |
| FTC14 | TOPAZ1 | chr3:44285086 | A | T | NM_001145030:c.A1088T:p.N363I | Missense |  |  |  |  |
| FTC14 | ABHD6 | chr3:58271093 | C | T | NM_020676:c.C750T:p.I250I | Silent |  |  |  |  |
| FTC14 | PHACTR1 | chr6:13273148 | G | T |  | Splicing |  |  |  |  |
| FTC14 | TTPA | chr8:63985561 | C | T | NM_000370:c.G291A:p.K97K | Silent |  |  |  |  |
| FTC14 | MUC6 | chr11:1018390 | C | T | NM_005961:c.G4411A:p.A1471T | Missense |  |  |  |  |
| FTC14 | MUC2 | chr11:1092890 | C | T | NM_002457:c.C4709T:p.T1570I | Missense |  |  |  |  |
| FTC14 | MYCBP2 | chr13:77718668 | G | A | NM_015057:c.C7215T:p.V2405V | Silent |  |  |  |  |
| FTC14 | ZNF263 | chr16:3333884 | C | T | NM_005741:c.C66T:p.C22C | Silent |  |  |  |  |
| FTC14 | SLC6A2 | chr16:55706070 | G | A | NM_001172502:c.G312A:p.P104P | Silent |  |  | COSM84345 |  |
| FTC14 | MYH4 | chr17:10357934 | G | T | NM_017533:c.C2629A:p.L877I | Missense |  |  |  |  |
| FTC14 | NF1 | chr17:29527441 | A | C | NM_000267:c.A890C:p.K297T | Missense | O | O |  | O |
| FTC14 | NKIRAS2 | chr17:40174501 | A | G | NM_001001349:c.A179G:p.Y60C | Missense |  |  |  |  |
| FTC14 | EZH1 | chr17:40858152 | T | C | NM_001991:c.A1712G:p.Q571R | Missense |  |  |  |  |
| FTC14 | TBX21 | chr17:45822271 | C | T | NM_013351:c.C1147T:p.Q383X | Nonsense |  |  |  |  |
| FTC14 | MUC16 | chr19:9066322 | G | C | NM_024690:c.C21124G:p.Q7042E | Missense |  |  |  |  |
| FTC14 | DMRTC2 | chr19:42352915 | C | A | NM_001040283:c.C500A:p.S167Y | Missense |  |  |  |  |
| FTC14 | ZBP1 | chr20:56188174 | G | A | NM_001160419:c.C715T:p.P239S | Missense |  |  |  |  |
| FTC14 | ARSA | chr22:51063717 | C | G | NM_000487:c.G1386C:p.Q462H | Missense |  |  |  |  |
| FTC01 | STIL | chr1:47728748 | C | T | NM_001048166:c.G2656A:p.V886M | Missense |  |  |  |  |
| FTC01 | NRAS | chr1:115256529 | T | C | NM_002524:c.A182G:p.Q61R | Missense | O | O | COSM584 | O |
| FTC01 | TRIML1 | chr4:189068196 | G | T | NM_178556:c.G1077T:p.K359N | Missense |  |  |  |  |
| FTC01 | IGF2BP3 | chr7:23353160 | A | G | NM_006547:c.T1508C:p.I503T | Missense |  |  |  |  |
| FTC01 | IGF2BP3 | chr7:23353231 | A | G | NM_006547:c.T1437C:p.F479F | Silent |  |  |  |  |
| FTC01 | PCLO | chr7:82579801 | G | T | NM_014510:c.C10103A:p.P3368Q | Missense |  |  |  |  |
| FTC01 | KRTAP5-4 | chr11:1642976 | A | C | NM_001012709:c.T348G:p.G116G | Silent |  |  |  |  |
| FTC01 | TFDP3 | chrX:132352188 | C | T | NM_016521:c.G100A:p.V34M | Missense |  |  | COSM1145563 |  |
| FTC02 | OXCT2 | chr1:40236128 | T | C | NM_022120:c.A800G:p.D267G | Missense |  |  | COSM3930778 |  |
| FTC02 | CELSR2 | chr1:109794384 | G | A | NM_001408:c.G1683A:p.W561X | Nonsense |  |  |  |  |
| FTC02 | NRAS | chr1:115256530 | G | T | NM_002524:c.C181A:p.Q61K | Missense | O | O | COSM580 | O |
| FTC02 | CAMK1G | chr1:209785148 | C | T | NM_020439:c.C927T:p.N309N | Silent |  |  |  |  |
| FTC02 | ADD1 | chr4:2901011 | G | T | NM_001119:c.G1010T:p.G337V | Missense |  |  |  |  |
| FTC02 | ADD1 | chr4:2901013 | C | G | NM_001119:c.C1012G:p.P338A | Missense |  |  |  |  |
| FTC02 | MGAM | chr7:141796215 | T | C | NM_004668:c.T5004C:p.R1668R | Silent |  |  |  |  |
| FTC02 | MUC2 | chr11:1092885 | G | A | NM_002457:c.G4704A:p.T1568T | Silent |  |  |  |  |
| FTC02 | MMP19 | chr12:56233376 | G | A | NM_002429:c.C670T:p.R224X | Nonsense |  |  |  |  |
| FTC02 | FMN1 | chr15:33359987 | G | A | NM_001103184:c.C99T:p.F33F | Silent |  |  |  |  |
| FTC02 | MXRA5 | chrX:3238765 | C | A | NM_015419:c.G4961T:p.G1654V | Missense |  |  |  |  |
| FTC03 | NRAS | chr1:115256530 | G | T | NM_002524:c.C181A:p.Q61K | Missense | O | O | COSM580 | O |
| FTC03 | MNDA | chr1:158815762 | C | A | NM_002432:c.C956A:p.T319K | Missense | O |  |  |  |
| FTC03 | OR10J5 | chr1:159505526 | G | T | NM_001004469:c.C272A:p.P91H | Missense |  |  |  |  |
| FTC03 | MOGS | chr2:74690478 | C | T | NM_006302:c.G615A:p.L205L | Silent |  |  |  |  |
| FTC03 | LRP2 | chr2:170003332 | C | T | NM_004525:c.G12728A:p.R4243Q | Missense |  |  | COSM441445 |  |
| FTC03 | TRIP12 | chr2:230643230 | C | T | NM_001284216:c.G4248A:p.K1416K | Silent |  |  |  |  |
| FTC03 | RPS3A | chr4:152024138 | A | C | NM_001006:c.A470C:p.Q157P | Missense |  |  | COSM328158 | O |
| FTC03 | PNLDC1 | chr6:160230107 | C | T | NM_001271862:c.C700T:p.R234X | Nonsense |  |  |  |  |
| FTC03 | MGAM | chr7:141796215 | T | C | NM_004668:c.T5004C:p.R1668R | Silent |  |  |  |  |
| FTC03 | CNTNAP2 | chr7:146829390 | C | T | NM_014141:c.C1137T:p.N379N | Silent |  |  | COSM1329992 |  |
| FTC03 | TUB | chr11:8118827 | A | G | NM_177972:c.A740G:p.E247G | Missense |  |  |  |  |
| FTC03 | ATXN3 | chr14:92537353 | C | CGCTGCTGCTGCT | NM_001164782:c.69_69delinsAGCAGCAGCAGCG | Nonframeshift |  |  |  |  |
| FTC03 | ANKRD40 | chr17:48784988 | G | A | NM_052855:c.C28T:p.Q10X | Nonsense |  |  |  |  |
| FTC03 | C22orf42 | chr22:32555158 | G | C | NM_001010859:c.C45G:p.L15L | Silent |  |  |  |  |
| FTC04 | HCN3 | chr1:155253896 | C | T | NM_020897:c.C840T:p.P280P | Silent |  |  |  |  |
| FTC04 | RGSL1 | chr1:182443028 | C | T | NM_001137669:c.C782T:p.P261L | Missense |  |  |  |  |
| FTC04 | FAM157A | chr3:197894694 | G | A | NM_001145248:c.G1036A:p.A346T | Missense |  |  |  |  |
| FTC04 | ZDHHC11 | chr5:840644 | G | C | NM_024786:c.C750G:p.H250Q | Missense |  |  |  |  |
| FTC04 | ADCY2 | chr5:7414735 | C | T | NM_020546:c.C260T:p.A87V | Missense |  |  | COSM215986 |  |
| FTC04 | METTL2B | chr7:128119351 | T | C | NM_018396:c.T342C:p.D114D | Silent |  |  |  |  |
| FTC04 | EYA1 | chr8:72267024 | G | T | NM_172058:c.C117A:p.G39G | Silent |  |  |  |  |
| FTC04 | SRPRA | chr11:126136799 | G | T | NM_001177842:c.C461A:p.A154D | Missense |  |  |  |  |
| FTC04 | NAB2 | chr12:57486945 | C | T | NM_005967:c.C1243T:p.L415L | Silent |  |  |  |  |
| FTC04 | ATXN3 | chr14:92537354 | C | CCTGCTGCTGCTGCTG | NM_001164782:c.68_68delinsCAGCAGCAGCAGCAGG | Nonframeshift |  |  |  |  |
| FTC04 | NDN | chr15:23932103 | G | T | NM_002487:c.C262A:p.P88T | Missense |  |  |  |  |
| FTC04 | ARIH1 | chr15:72873082 | C | A | NM_005744:c.C1226A:p.A409E | Missense |  |  |  |  |
| FTC04 | BNC1 | chr15:83933349 | G | A | NM_001301206:c.C633T:p.L211L | Silent |  |  |  |  |
| FTC04 | CLEC18B | chr16:74447025 | T | C | NM_001011880:c.A586G:p.I196V | Missense |  |  |  |  |
| FTC04 | MAP1LC3B | chr16:87436663 | A | G | NM_022818:c.A338G:p.Y113C | Missense |  |  | COSM3717123 |  |
| FTC04 | NPEPPS | chr17:45664677 | C | T | NM_006310:c.C1062T:p.L354L | Silent |  |  | COSM706022 |  |
| FTC05 | TP53BP2 | chr1:223989955 | G | C | NM_001031685:c.C1088G:p.P363R | Missense |  |  |  | O |
| FTC05 | ENAH | chr1:225702599 | A | T | NM_001008493:c.T917A:p.I306N | Missense |  |  |  |  |
| FTC05 | REEP1 | chr2:86444228 | C | T | NM_001164731:c.G520A:p.A174T | Missense |  |  |  |  |
| FTC05 | ACMSD | chr2:135630066 | G | A | NM_138326:c.G704A:p.R235K | Missense |  |  |  |  |
| FTC05 | HEG1 | chr3:124732191 | G | C | NM_020733:c.C2232G:p.T744T | Silent |  |  |  |  |
| FTC05 | LAMA4 | chr6:112453957 | C | A | NM_001105206:c.G3832T:p.G1278W | Missense |  |  |  | O |
| FTC05 | ZNF425 | chr7:148802310 | C | A | NM_001001661:c.G653T:p.C218F | Missense |  |  | COSM1234040 |  |
| FTC05 | INTS8 | chr8:95892426 | A | G | NM_017864:c.A2952G:p.K984K | Silent |  |  |  |  |
| FTC05 | GPR158 | chr10:25464635 | C | A | NM_020752:c.C286A:p.R96R | Silent |  |  |  |  |
| FTC05 | MUC2 | chr11:1093368 | G | A | NM_002457:c.G5187A:p.T1729T | Silent |  |  |  |  |
| FTC05 | OR5M9 | chr11:56230302 | G | C | NM_001004743:c.C576G:p.I192M | Missense |  |  |  |  |
| FTC05 | DHCR7 | chr11:71152330 | G | A | NM_001163817:c.C569T:p.A190V | Missense |  |  |  |  |
| FTC05 | FAM216A | chr12:110925733 | C | T | NM_013300:c.C688T:p.L230L | Silent |  |  |  |  |
| FTC05 | CDAN1 | chr15:43016807 | G | A | NM_138477:c.C3566T:p.A1189V | Missense |  |  |  |  |
| FTC05 | TCF12 | chr15:57524917 | C | A | NM_207040:c.C323A:p.P108H | Missense | O |  |  | O |
| FTC05 | CDH11 | chr16:65006806 | C | T |  | Splicing |  |  | COSM1177881 |  |
| FTC05 | PLD2 | chr17:4720539 | G | A | NM_001243108:c.G1800A:p.Q600Q | Silent |  |  |  |  |
| FTC05 | KRTAP4-8 | chr17:39254054 | A | T | NM_031960:c.T283A:p.C95S | Missense |  |  |  |  |
| FTC05 | FERMT1 | chr20:6068488 | A | G | NM_017671:c.T1307C:p.F436S | Missense |  |  |  |  |
| FTC05 | ZNF81 | chrX:47775465 | G | A | NM_007137:c.G1420A:p.D474N | Missense |  |  |  |  |
| FTC06 | AHCYL1 | chr1:110558145 | T | A | NM_001242673:c.T633A:p.G211G | Silent |  |  |  |  |
| FTC06 | BIRC6 | chr2:32626268 | G | A | NM_016252:c.G1072A:p.V358M | Missense |  |  |  |  |
| FTC06 | ABCB11 | chr2:169828364 | A | T | NM_003742:c.T1631A:p.L544Q | Missense |  |  |  |  |
| FTC06 | TTN | chr2:179396456 | A | T | NM_003319:c.T77691A:p.I25897I | Silent |  |  |  |  |
| FTC06 | DYTN | chr2:207530665 | T | A | NM_001093730:c.A1069T:p.R357W | Missense |  |  |  |  |
| FTC06 | PIK3R4 | chr3:130463422 | T | C | NM_014602:c.A641G:p.Y214C | Missense |  |  |  |  |
| FTC06 | WASF1 | chr6:110426687 | C | A | NM_001024936:c.G636T:p.E212D | Missense |  |  |  |  |
| FTC06 | REV3L | chr6:111689142 | C | A | NM_002912:c.G5849T:p.R1950L | Missense |  |  |  |  |
| FTC06 | MAP3K5 | chr6:136944078 | T | A | NM_005923:c.A2058T:p.L686F | Missense |  |  |  |  |
| FTC06 | IGF2BP3 | chr7:23353160 | A | G | NM_006547:c.T1508C:p.I503T | Missense |  |  |  |  |
| FTC06 | IGF2BP3 | chr7:23353231 | A | G | NM_006547:c.T1437C:p.F479F | Silent |  |  |  |  |
| FTC06 | CTAGE4 | chr7:143882747 | A | G | NM_198495:c.A2151G:p.R717R | Silent |  |  |  |  |
| FTC06 | MUC2 | chr11:1092885 | G | C | NM_002457:c.G4704C:p.T1568T | Silent |  |  |  |  |
| FTC06 | GPR180 | chr13:95271757 | G | T | NM_180989:c.G722T:p.G241V | Missense |  |  |  |  |
| FTC06 | AGBL1 | chr15:86807910 | T | A | NM_152336:c.T1370A:p.L457Q | Missense |  |  |  |  |
| FTC06 | ITGAM | chr16:31282370 | A | G | NM_000632:c.A523G:p.T175A | Missense |  |  |  |  |
| FTC06 | CNOT1 | chr16:58589312 | T | A | NM_001265612:c.A2719T:p.I907L | Missense | O |  |  | O |
| FTC06 | MYBBP1A | chr17:4446021 | C | G | NM_001105538:c.G2908C:p.D970H | Missense |  |  |  |  |
| FTC06 | SUGP1 | chr19:19416723 | G | A | NM_172231:c.C473T:p.P158L | Missense |  |  |  |  |
| FTC06 | FPR1 | chr19:52249863 | G | A | NM_002029:c.C385T:p.H129Y | Missense |  |  |  |  |
| FTC06 | KRTAP13-1 | chr21:31768624 | A | T | NM_181599:c.A220T:p.S74C | Missense |  |  |  |  |
| FTC07 | ARHGEF11 | chr1:156907084 | A | C | NM_014784:c.T4277G:p.F1426C | Missense |  |  |  |  |
| FTC07 | MYT1L | chr2:1843073 | G | A | NM_015025:c.C2922T:p.T974T | Silent |  |  |  |  |
| FTC07 | CHN1 | chr2:175677096 | G | A | NM_001206602:c.C452T:p.A151V | Missense |  |  |  |  |
| FTC07 | MAML3 | chr4:140811081 | C | CTGT | NM_018717:c.1509_1509delinsACAG | Nonframeshift |  |  |  |  |
| FTC07 | AGGF1 | chr5:76332463 | C | A | NM_018046:c.C599A:p.A200E | Missense |  |  |  |  |
| FTC07 | NRCAM | chr7:107790402 | T | G | NM_005010:c.A3505C:p.S1169R | Missense |  |  |  | O |
| FTC07 | IFIT2 | chr10:91066289 | G | T | NM_001547:c.G576T:p.Q192H | Missense |  |  |  |  |
| FTC07 | DTX4 | chr11:58956785 | A | G | NM_001300727:c.A830G:p.Q277R | Missense |  |  |  | O |
| FTC07 | CS | chr12:56676244 | C | T | NM_004077:c.G548A:p.R183Q | Missense |  |  | COSM224433 |  |
| FTC07 | SACS | chr13:23908969 | T | G | NM_001278055:c.A8605C:p.N2869H | Missense |  |  |  |  |
| FTC07 | METTL16 | chr17:2371165 | T | C | NM_024086:c.A475G:p.K159E | Missense |  |  |  | O |
| FTC07 | PSMD3 | chr17:38146102 | A | C | NM_002809:c.A797C:p.Q266P | Missense |  |  |  |  |
| FTC07 | NPEPPS | chr17:45664677 | C | T | NM_006310:c.C1062T:p.L354L | Silent |  |  | COSM706022 |  |
| FTC07 | BRIP1 | chr17:59821931 | G | A | NM_032043:c.C2119T:p.R707C | Missense |  | O | COSM2793788 | O |
| FTC07 | MUC16 | chr19:9073581 | A | T | NM_024690:c.T13865A:p.L4622H | Missense |  |  |  |  |
| FTC07 | C19orf12 | chr19:30199315 | A | G | NM_001031726:c.T39C:p.T13T | Silent |  |  |  |  |
| FTC07 | PSG9 | chr19:43762539 | G | A | NM_001301709:c.C500T:p.S167F | Missense |  |  |  |  |
| FTC07 | MACROD2 | chr20:16025232 | G | T | NM_001033087:c.G543T:p.Q181H | Missense |  |  |  |  |
| FTC07 | ZGPAT | chr20:62340111 | G | A | NM_001083113:c.G179A:p.S60N | Missense |  |  |  |  |
| FTC08 | FPGT | chr1:74664006 | C | G | NM_001112808:c.C83G:p.A28G | Missense |  |  |  |  |
| FTC08 | ATP1A4 | chr1:160147405 | G | T | NM_001001734:c.G95T:p.W32L | Missense |  |  |  | O |
| FTC08 | TUBA3D | chr2:132236921 | G | A | NM_080386:c.G267A:p.P89P | Silent |  |  |  |  |
| FTC08 | MROH2A | chr2:234704883 | C | T | NM_001287395:c.C1185T:p.F395F | Silent |  |  |  |  |
| FTC08 | ABCF3 | chr3:183907381 | C | G | NM_018358:c.C1150G:p.R384G | Missense |  |  |  |  |
| FTC08 | SH3TC2 | chr5:148407891 | G | T | NM_024577:c.C1404A:p.A468A | Silent |  |  |  |  |
| FTC08 | NMUR2 | chr5:151777678 | C | G | NM_020167:c.G754C:p.D252H | Missense |  |  |  |  |
| FTC08 | TXNDC5 | chr6:7899862 | C | G | NM_001145549:c.G142C:p.D48H | Missense |  |  |  | O |
| FTC08 | TMEM200A | chr6:130762192 | A | G | NM_001258276:c.A625G:p.T209A | Missense |  |  |  |  |
| FTC08 | GRM1 | chr6:146351138 | C | A | NM_001278064:c.C485A:p.P162H | Missense |  |  |  | O |
| FTC08 | RSPH3 | chr6:159398659 | C | T | NM_031924:c.G1594A:p.E532K | Missense |  |  |  |  |
| FTC08 | ARHGAP22 | chr10:49661392 | T | C | NM_001256026:c.A673G:p.N225D | Missense |  |  |  |  |
| FTC08 | CDH23 | chr10:73377212 | T | A | NM_001171932:c.T1196A:p.L399Q | Missense |  |  |  |  |
| FTC08 | PML | chr15:74336743 | G | T | NM_033238:c.G2043T:p.G681G | Silent |  |  |  |  |
| FTC08 | UBE2Q2 | chr15:76183280 | A | T | NM_001284382:c.A849T:p.S283S | Silent |  |  |  |  |
| FTC08 | RRN3 | chr16:15168670 | G | T | NM_001301064:c.C817A:p.L273I | Missense |  |  |  |  |
| FTC08 | NPIPB5 | chr16:22545785 | C | T | NM_001135865:c.C1481T:p.T494I | Missense |  |  |  | O |
| FTC08 | KRTAP4-8 | chr17:39254054 | A | T | NM_031960:c.T283A:p.C95S | Missense |  |  |  |  |
| FTC08 | KRTAP9-8 | chr17:39394445 | C | T | NM_031963:c.C142T:p.R48C | Missense |  |  |  |  |
| FTC08 | DMD | chrX:32360345 | G | T | NM_004011:c.C1771A:p.Q591K | Missense |  |  |  |  |
| FTC08 | ATP6AP2 | chrX:40448260 | T | A | NM_005765:c.T60A:p.S20R | Missense | O |  |  |  |
| FTC08 | ABCD1 | chrX:152991234 | C | A | NM_000033:c.C513A:p.Y171X | Nonsense |  |  |  |  |
| FTC09 | CA6 | chr1:9005954 | C | A | NM_001215:c.C9A:p.A3A | Silent |  |  |  |  |
| FTC09 | WDTC1 | chr1:27632721 | T | A | NM_001276252:c.T1881A:p.A627A | Silent |  |  |  |  |
| FTC09 | TSSK3 | chr1:32829655 | T | C | NM_052841:c.T605C:p.V202A | Missense |  |  |  |  |
| FTC09 | MAP4K3 | chr2:39553068 | G | A | NM_001270425:c.C697T:p.P233S | Missense | O |  |  | O |
| FTC09 | ALS2CR11 | chr2:202412236 | C | A | NM_001168216:c.G1075T:p.D359Y | Missense |  |  |  |  |
| FTC09 | IL31RA | chr5:55195942 | C | A | NM_001242636:c.C994A:p.P332T | Missense |  |  |  |  |
| FTC09 | NEDD9 | chr6:11190915 | G | T | NM_001271033:c.C740A:p.P247Q | Missense |  |  |  |  |
| FTC09 | CDC40 | chr6:110539004 | C | A | NM_015891:c.C1088A:p.T363K | Missense |  |  |  | O |
| FTC09 | IGF2BP3 | chr7:23353160 | A | G | NM_006547:c.T1508C:p.I503T | Missense |  |  |  |  |
| FTC09 | IGF2BP3 | chr7:23353231 | A | G | NM_006547:c.T1437C:p.F479F | Silent |  |  |  |  |
| FTC09 | BRAF | chr7:140453134 | T | C | NM_004333:c.A1801G:p.K601E | Missense | O | O | COSM478 | O |
| FTC09 | TG | chr8:133880467 | C | T | NM_003235:c.C175T:p.Q59X | Nonsense |  |  |  |  |
| FTC09 | MUC2 | chr11:1088847 | G | T | NM_002457:c.G3632T:p.C1211F | Missense |  |  |  |  |
| FTC09 | CNGA4 | chr11:6261310 | G | A | NM_001037329:c.G286A:p.G96S | Missense |  |  |  |  |
| FTC09 | ARHGAP20 | chr11:110485344 | C | A | NM_001258415:c.G502T:p.D168Y | Missense |  |  |  | O |
| FTC09 | OR4D5 | chr11:123810668 | G | A | NM_001001965:c.G345A:p.L115L | Silent |  |  |  |  |
| FTC09 | TMTC2 | chr12:83289939 | G | A | NM_152588:c.G997A:p.V333I | Missense |  |  |  |  |
| FTC09 | SULT1A2 | chr16:28604854 | C | T | NM_001054:c.G408A:p.A136A | Silent |  |  |  |  |
| FTC09 | MYH8 | chr17:10298528 | C | T | NM_002472:c.G4884A:p.M1628I | Missense |  |  |  |  |
| FTC09 | ZNF614 | chr19:52519738 | A | G | NM_025040:c.T1113C:p.Y371Y | Silent |  |  |  |  |
| FTC09 | DGCR8 | chr22:20077562 | G | T | NM_001190326:c.G1087T:p.E363X | Nonsense |  |  |  |  |
| FTC09 | DGCR8 | chr22:20082257 | T | G | NM_001190326:c.T1628G:p.L543R | Missense |  |  |  | O |
| FTC09 | TCF20 | chr22:42609211 | AT | A | NM_181492:c.2100_2101T | Frameshift |  |  |  |  |
| FTC09 | MID2 | chrX:107159239 | A | G | NM_012216:c.A1081G:p.M361V | Missense |  |  |  |  |
| FTC10 | EPHA2 | chr1:16461637 | C | G | NM_004431:c.G1476C:p.V492V | Silent | O |  |  |  |
| FTC10 | ARID1A | chr1:27100174 | T | A | NM_006015:c.T3970A:p.Y1324N | Missense | O | O |  |  |
| FTC10 | CYP4Z1 | chr1:47548113 | G | A | NM_178134:c.G472A:p.E158K | Missense |  |  |  |  |
| FTC10 | NRAS | chr1:115256529 | T | C | NM_002524:c.A182G:p.Q61R | Missense | O | O | COSM584 | O |
| FTC10 | IGSF3 | chr1:117158972 | A | G | NM_001542:c.T151C:p.S51P | Missense |  |  |  |  |
| FTC10 | IGSF3 | chr1:117158983 | T | G | NM_001542:c.A140C:p.Y47S | Missense |  |  |  |  |
| FTC10 | MGAT4B | chr5:179225937 | CTT | C | NM_054013:c.1377_1379G | Frameshift |  |  |  |  |
| FTC10 | COL10A1 | chr6:116442476 | G | T | NM_000493:c.C803A:p.A268D | Missense |  |  |  |  |
| FTC10 | TBP | chr6:170871058 | G | A | NM_001172085:c.G174A:p.Q58Q | Silent |  |  |  |  |
| FTC10 | ZPBP | chr7:50070741 | C | T | NM_001159878:c.G650A:p.R217H | Missense |  |  | COSM1488596 |  |
| FTC10 | TG | chr8:133898784 | C | A | NM_003235:c.C1167A:p.F389L | Missense |  |  |  |  |
| FTC10 | CDHR5 | chr11:621437 | A | T | NM_001171968:c.T526A:p.S176T | Missense |  |  |  |  |
| FTC10 | MUC2 | chr11:1092987 | G | T | NM_002457:c.G4806T:p.Q1602H | Missense |  |  |  |  |
| FTC10 | MUC2 | chr11:1093314 | A | T | NM_002457:c.A5133T:p.P1711P | Silent |  |  |  |  |
| FTC10 | RBM19 | chr12:114395708 | C | A | NM_001146698:c.G719T:p.S240I | Missense |  |  |  | O |
| FTC10 | ADAM21 | chr14:70924335 | C | T | NM_003813:c.C119T:p.P40L | Missense |  |  |  |  |
| FTC10 | CEMIP | chr15:81173448 | C | CA | NM_018689:c.588_588delinsCA | Frameshift |  |  |  |  |
| FTC10 | RRN3 | chr16:15159152 | C | T | NM_001301064:c.G1540A:p.A514T | Missense |  |  |  |  |
| FTC10 | FHOD1 | chr16:67273336 | C | G | NM_013241:c.G223C:p.V75L | Missense |  |  |  |  |
| FTC10 | SLC35G6 | chr17:7386091 | C | A | NM_001102614:c.C788A:p.A263D | Missense |  |  |  |  |
| FTC10 | CASK | chrX:41469158 | T | A | NM_001126055:c.A1136T:p.D379V | Missense |  |  |  | O |
| FTC10 | MED12 | chrX:70347199 | G | A | NM_005120:c.G2863A:p.V955M | Missense | O | O |  |  |
| FTC10 | PGAM4 | chrX:77224847 | C | A | NM_001029891:c.G289T:p.G97C | Missense |  |  |  |  |
| FTC11 | TMEM51 | chr1:15541632 | A | G | NM_001136217:c.A49G:p.I17V | Missense |  |  |  |  |
| FTC11 | SLAMF7 | chr1:160718255 | C | A | NM_001282588:c.C327A:p.S109R | Missense |  |  |  |  |
| FTC11 | ASB18 | chr2:237172816 | G | A | NM_212556:c.C173T:p.P58L | Missense |  |  |  |  |
| FTC11 | KCNMB3 | chr3:178957797 | T | A | NM_001163677:c.A509T:p.E170V | Missense |  |  |  |  |
| FTC11 | MAML3 | chr4:140811081 | C | CTGT | NM_018717:c.1509_1509delinsACAG | Nonframeshift |  |  |  |  |
| FTC11 | TMEM14B | chr6:10756712 | C | T | NM_001127711:c.C204T:p.A68A | Silent |  |  |  |  |
| FTC11 | ALDH8A1 | chr6:135253935 | G | A | NM_001193480:c.C678T:p.V226V | Silent |  |  |  |  |
| FTC11 | PHF14 | chr7:11076620 | A | G | NM_014660:c.A1882G:p.M628V | Missense |  |  |  |  |
| FTC11 | H2AFV | chr7:44874131 | A | G | NM_201436:c.T278C:p.I93T | Missense |  |  |  |  |
| FTC11 | ZNF746 | chr7:149190046 | G | A | NM_001163474:c.C424T:p.P142S | Missense |  |  | COSM1289202 |  |
| FTC11 | PKHD1L1 | chr8:110460490 | G | A | NM_177531:c.G5895A:p.V1965V | Silent |  |  |  |  |
| FTC11 | HRAS | chr11:533874 | T | C | NM_001130442:c.A182G:p.Q61R | Missense | O | O | COSM499;COSM244958 | O |
| FTC11 | ABCC8 | chr11:17464838 | G | A | NM_000352:c.C1354T:p.L452F | Missense |  |  |  | O |
| FTC11 | USP35 | chr11:77924706 | G | A | NM_020798:c.G2904A:p.E968E | Silent |  |  |  |  |
| FTC11 | ZCCHC8 | chr12:122958748 | G | A | NM_017612:c.C1420T:p.P474S | Missense |  |  |  |  |
| FTC11 | NUFIP1 | chr13:45523879 | T | C | NM_012345:c.A1116G:p.S372S | Silent |  |  | COSM403671 |  |
| FTC11 | PCNX | chr14:71476390 | A | T | NM_014982:c.A2669T:p.D890V | Missense |  |  |  |  |
| FTC11 | SULT1A2 | chr16:28604854 | C | T | NM_001054:c.G408A:p.A136A | Silent |  |  |  |  |
| FTC11 | TRAPPC8 | chr18:29470724 | C | T | NM_014939:c.G1702A:p.G568S | Missense |  |  |  | O |
| FTC11 | RNF152 | chr18:59483178 | G | A | NM_173557:c.C519T:p.I173I | Silent |  |  |  |  |
| FTC11 | LILRB1 | chr19:55144208 | G | A | NM_001081637:c.G955A:p.A319T | Missense |  |  |  |  |
| FTC11 | SON | chr21:34925385 | G | T | NM_001291411:c.G3848T:p.C1283F | Missense |  |  |  |  |
| FTC11 | EIF1AX | chrX:20148727 | T | A |  | Splicing | O |  |  |  |
| FTC11 | STAG2 | chrX:123184131 | A | C | NM_006603:c.A989C:p.K330T | Missense | O | O |  | O |
| FTC12 | HNRNPR | chr1:23667455 | TC | T | NM_001102398:c.46_47A | Frameshift |  |  |  |  |
| FTC12 | PPP1R15B | chr1:204379575 | T | C | NM_032833:c.A965G:p.Y322C | Missense |  |  |  |  |
| FTC12 | CCDC74A | chr2:132290441 | G | A | NM_001258305:c.G688A:p.G230S | Missense |  |  |  |  |
| FTC12 | ZKSCAN7 | chr3:44598788 | C | T | NM_001288590:c.C249T:p.L83L | Silent |  |  |  |  |
| FTC12 | OR5H6 | chr3:97983602 | T | C | NM_001005479:c.T474C:p.I158I | Silent |  |  | COSM1717715 |  |
| FTC12 | SH3RF1 | chr4:170190184 | G | A | NM_020870:c.C180T:p.V60V | Silent |  |  |  |  |
| FTC12 | CDH6 | chr5:31267591 | A | G | NM_004932:c.A11G:p.Y4C | Missense |  |  |  |  |
| FTC12 | EDIL3 | chr5:83360656 | C | T | NM_001278642:c.G785A:p.R262H | Missense |  |  | COSM3828563 |  |
| FTC12 | LATS1 | chr6:149983004 | T | C | NM_004690:c.A3254G:p.D1085G | Missense |  |  |  | O |
| FTC12 | PLG | chr6:161137725 | G | A | NM_000301:c.G717A:p.R239R | Silent |  |  |  |  |
| FTC12 | CDHR3 | chr7:105664907 | C | T | NM_001301161:c.C1893T:p.G631G | Silent |  |  |  |  |
| FTC12 | TRPA1 | chr8:72938260 | G | A | NM_007332:c.C2986T:p.R996C | Missense |  |  |  |  |
| FTC12 | PTGS1 | chr9:125133517 | C | A | NM_000962:c.C60A:p.P20P | Silent | O |  |  |  |
| FTC12 | LIPF | chr10:90438398 | A | T | NM_001198828:c.A1058T:p.Y353F | Missense |  |  |  |  |
| FTC12 | CH25H | chr10:90966365 | G | T | NM_003956:c.C685A:p.P229T | Missense |  |  |  |  |
| FTC12 | CH25H | chr10:90966366 | C | T | NM_003956:c.G684A:p.V228V | Silent |  |  |  |  |
| FTC12 | OR5D13 | chr11:55540919 | G | A | NM_001001967:c.G6A:p.M2I | Missense |  |  |  |  |
| FTC12 | MPEG1 | chr11:58979309 | A | G | NM_001039396:c.T1030C:p.F344L | Missense |  |  |  |  |
| FTC12 | USP35 | chr11:77921177 | G | C | NM_020798:c.G2276C:p.R759P | Missense |  |  |  | O |
| FTC12 | CUL5 | chr11:107969223 | A | G | NM_003478:c.A2115G:p.E705E | Silent |  |  |  |  |
| FTC12 | DZIP1 | chr13:96277101 | A | G | NM_014934:c.T893C:p.V298A | Missense |  |  |  |  |
| FTC12 | MYO16 | chr13:109550375 | C | T | NM_001198950:c.C1671T:p.I557I | Silent |  |  |  |  |
| FTC12 | SLC12A6 | chr15:34529662 | T | C | NM_001042497:c.A2847G:p.L949L | Silent |  |  |  |  |
| FTC12 | ACAN | chr15:89392810 | G | A | NM_001135:c.G1874A:p.G625D | Missense |  |  |  | O |
| FTC12 | SRL | chr16:4253192 | A | C | NM_001098814:c.T234G:p.N78K | Missense |  |  |  |  |
| FTC12 | SPDYE4 | chr17:8659749 | G | T | NM_001128076:c.C346A:p.P116T | Missense |  |  | COSM986361 |  |
| FTC12 | CDC27 | chr17:45234350 | C | T | NM_001293091:c.G588A:p.Q196Q | Silent | O |  |  |  |
| FTC12 | CDC27 | chr17:45234367 | A | T | NM_001293091:c.T571A:p.S191T | Missense | O |  |  |  |
| FTC12 | CDC27 | chr17:45234386 | G | T | NM_001293091:c.C552A:p.V184V | Silent | O |  |  |  |
| FTC12 | PSG9 | chr19:43772276 | C | T | NM_001301707:c.G90A:p.P30P | Silent |  |  |  |  |
| FTC12 | ZNF180 | chr19:44981081 | A | C | NM_001278508:c.T1542G:p.H514Q | Missense |  |  |  |  |
| FTC12 | NCOA3 | chr20:46279839 | G | A | NM_001174087:c.G3762A:p.Q1254Q | Silent |  |  |  |  |
| FTC12 | SPANXN1 | chrX:144337223 | C | A | NM_001009614:c.C108A:p.P36P | Silent |  |  |  |  |
| FTC13 | HIVEP3 | chr1:41978896 | G | A | NM_001127714:c.C5996T:p.P1999L | Missense |  |  |  |  |
| FTC13 | OR10T2 | chr1:158368889 | C | T | NM_001004475:c.G368A:p.R123H | Missense |  |  |  |  |
| FTC13 | FMO1 | chr1:171254411 | A | G | NM_001282692:c.A1339G:p.I447V | Missense |  |  |  |  |
| FTC13 | RYR2 | chr1:237732446 | C | A | NM_001035:c.C3425A:p.A1142D | Missense |  |  |  |  |
| FTC13 | STRN | chr2:37113858 | C | G |  | Splicing |  |  |  |  |
| FTC13 | TTN | chr2:179419330 | A | T | NM_003319:c.T61549A:p.S20517T | Missense |  |  |  |  |
| FTC13 | ANKZF1 | chr2:220100211 | G | A | NM_001282792:c.G1077A:p.R359R | Silent |  |  |  |  |
| FTC13 | COL4A4 | chr2:227895226 | G | A | NM_000092:c.C3906T:p.P1302P | Silent |  |  |  |  |
| FTC13 | MUC20 | chr3:195452960 | G | A | NM_001282506:c.G1486A:p.V496I | Missense | O |  |  |  |
| FTC13 | GABRB1 | chr4:47163439 | T | G | NM_000812:c.T414G:p.N138K | Missense |  |  |  |  |
| FTC13 | MCM3 | chr6:52137144 | A | C | NM_001270472:c.T1812G:p.Y604X | Nonsense | O |  |  |  |
| FTC13 | MAP3K5 | chr6:136904819 | T | C | NM_005923:c.A3285G:p.R1095R | Silent |  |  |  |  |
| FTC13 | FOXK1 | chr7:4780516 | C | T | NM_001037165:c.C608T:p.S203L | Missense |  |  |  |  |
| FTC13 | MKLN1 | chr7:131130612 | C | G | NM_013255:c.C1475G:p.S492C | Missense |  |  |  |  |
| FTC13 | KMT2C | chr7:151945256 | G | A | NM_170606:c.C2263T:p.Q755X | Nonsense |  | O | COSM3733336 |  |
| FTC13 | OPLAH | chr8:145108284 | G | T | NM_017570:c.C2699A:p.A900D | Missense |  |  |  |  |
| FTC13 | CCDC183 | chr9:139698997 | G | T | NM_001039374:c.G710T:p.R237L | Missense |  |  |  |  |
| FTC13 | CTBP2 | chr10:126727569 | C | T | NM_001083914:c.G55A:p.E19K | Missense |  |  |  |  |
| FTC13 | CCKBR | chr11:6290988 | C | G | NM_176875:c.C241G:p.L81V | Missense |  |  |  |  |
| FTC13 | ZBTB3 | chr11:62520296 | C | A | NM_024784:c.G991T:p.A331S | Missense |  |  |  |  |
| FTC13 | PAK1 | chr11:77066841 | C | T | NM_001128620:c.G644A:p.R215Q | Missense |  |  |  |  |
| FTC13 | LRRK2 | chr12:40704414 | T | C | NM_198578:c.T4499C:p.L1500P | Missense |  |  |  |  |
| FTC13 | KRT85 | chr12:52756203 | G | T | NM_001300810:c.C494A:p.A165D | Missense |  |  |  |  |
| FTC13 | CS | chr12:56676244 | C | T | NM_004077:c.G548A:p.R183Q | Missense |  |  | COSM224433 |  |
| FTC13 | FLVCR2 | chr14:76045856 | G | A | NM_017791:c.G541A:p.V181M | Missense |  |  |  |  |
| FTC13 | SCG5 | chr15:32972043 | C | T | NM_001144757:c.C303T:p.D101D | Silent |  |  |  |  |
| FTC13 | C15orf57 | chr15:40846274 | T | C | NM_001080791:c.A481G:p.T161A | Missense |  |  |  |  |
| FTC13 | CLN6 | chr15:68510982 | G | T | NM_017882:c.C90A:p.G30G | Silent |  |  |  |  |
| FTC13 | CSPG4 | chr15:75981385 | G | T | NM_001897:c.C2021A:p.A674D | Missense |  |  |  |  |
| FTC13 | CHSY1 | chr15:101717844 | C | T | NM_014918:c.G2158A:p.D720N | Missense |  |  |  |  |
| FTC13 | GALNS | chr16:88891188 | T | G | NM_000512:c.A1229C:p.E410A | Missense |  |  |  |  |
| FTC13 | POTEC | chr18:14513764 | C | T | NM_001137671:c.G1430A:p.R477Q | Missense |  |  |  |  |
| FTC13 | ZNF98 | chr19:22585603 | T | C | NM_001098626:c.A241G:p.T81A | Missense |  |  | COSM1129924;COSM1129923 |  |
| FTC13 | LRFN3 | chr19:36430885 | C | A | NM_024509:c.C558A:p.G186G | Silent |  |  |  |  |
| FTC13 | ZNF112 | chr19:44833896 | C | A | NM_013380:c.G414T:p.W138C | Missense |  |  |  |  |
| FTC13 | ZNF587 | chr19:58370938 | G | A | NM_001204817:c.G1155A:p.G385G | Silent |  |  |  |  |
| FTC13 | DDX27 | chr20:47849941 | A | T | NM_017895:c.A1223T:p.E408V | Missense |  |  |  |  |
| FTC13 | PHKA1 | chrX:71873308 | G | A | NM_001122670:c.C1114T:p.L372L | Silent |  |  |  |  |
| FTC13 | PHKA1 | chrX:71873309 | C | T | NM_001122670:c.G1113A:p.E371E | Silent |  |  |  |  |
| FTC13 | DOCK11 | chrX:117700592 | G | A | NM_144658:c.G927A:p.R309R | Silent |  |  |  |  |

* UCSC GRCh37/hg19

** Cancer Drivers Database (http://www.intogen.org/downloads)

† Cancer Gene Census (http://cancer.sanger.ac.uk/census)

‡ Cosmic (http://cancer.sanger.ac.uk/cosmic)

$ CHASM (http://www.cravat.us/)
