# Supplementary material for: Mutational burdens and evolutionary ages of thyroid follicular adenoma are comparable to those of follicular carcinoma
Source: Oncotarget. 2016 Sep 9;7(43):69638–48. doi: 10.18632/oncotarget.11922 (PMC5342504; doi:10.18632/oncotarget.11922)
Supplement: Supplementary file 3 [file oncotarget-07-69638-s003.docx]

**Table S4. Copy number alterations identified across 27 follicular tumors by array-CGH.**

| **Sample ID*** | **Genomic position** | **Event** | **Length** | **Cytoband** | **Cancer Gene Census**** |
| --- | --- | --- | --- | --- | --- |
| FTA02 | chr17:0-22,032,763 | CN Gain | 22,032,764 | p13.3 - p11.2 | YWHAE, USP6, TP53, PER1, GAS7, MAP2K4 |
| FTA02 | chr17:25,403,446-81,195,210 | CN Gain | 55,791,765 | q11.1 - q25.3 | NF1, SUZ12, TAF15, MLLT6, LASP1, CDK12, ERBB2, RARA, BRCA1, ETV4, COL1A1, HLF, MSI2, CLTC, BRIP1, CD79B, DDX5, PRKAR1A, SRSF2, CANT1, ASPSCR1 |
| FTA08 | chr16:0-34,081,660 | CN Gain | 34,081,661 | p13.3 - p11.2 | TSC2, CREBBP, CIITA, SOCS1, TNFRSF17, ERCC4, MYH11, PALB2, IL21R, FUS |
| FTA08 | chr19:0-24,340,741 | CN Loss | 24,340,742 | p13.3 - p12 | FSTL3, STK11, TCF3, GNA11, SH3GL1, MLLT1, DNM2, SMARCA4, LYL1, BRD4, TPM4, JAK3, ELL |
| FTA12 | chr22:16,486,086-51,304,566 | CN Loss | 34,818,481 | q11.1 - q13.33 | CLTCL1, BCR, SMARCB1, MN1, CHEK2, EWSR1, NF2, MYH9, PDGFB, MKL1, EP300 |
| FTC01 | chr22:16,486,086-51,304,566 | CN Loss | 34,818,481 | q11.1 - q13.33 | CLTCL1, BCR, SMARCB1, MN1, CHEK2, EWSR1, NF2, MYH9, PDGFB, MKL1, EP300 |
| FTC02 | chr22:16,486,086-51,304,566 | CN Loss | 34,818,481 | q11.1 - q13.33 | CLTCL1, BCR, SMARCB1, MN1, CHEK2, EWSR1, NF2, MYH9, PDGFB, MKL1, EP300 |
| FTC03 | chr22:16,486,086-51,304,566 | CN Loss | 34,818,481 | q11.1 - q13.33 | CLTCL1, BCR, SMARCB1, MN1, CHEK2, EWSR1, NF2, MYH9, PDGFB, MKL1, EP300 |
| FTC04 | chr22:16,486,086-51,304,566 | CN Loss | 34,818,481 | q11.1 - q13.33 | CLTCL1, BCR, SMARCB1, MN1, CHEK2, EWSR1, NF2, MYH9, PDGFB, MKL1, EP300 |
| FTC05 | chr14:22,451,012-22,970,589 | CN Gain | 519,578 | q11.2 |  |
| FTC05 | chr14:106,088,866-107,219,377 | CN Gain | 1,130,512 | q32.33 | IGH |
| FTC05 | chr20:0-21,487,627 | CN Gain | 21,487,628 | p13 - p11.22 |  |
| FTC07 | chr1:144,009,907-249,250,621 | CN Gain | 105,240,715 | q21.1 - q44 | PDE4DIP, BCL9, ARNT, TPM3, MUC1, PRCC, NTRK1, SDHC, FCGR2B, PBX1, ABL2, TPR, MDM4, ELK4, SLC45A3, H3F3A, FH |
| FTC07 | chr14:77,992,503-78,450,289 | CN Gain | 457,787 | q24.3 |  |
| FTC07 | chr16:48,889,715-52,830,326 | CN Loss | 3,940,612 | q12.1 - q12.2 | CYLD |
| FTC07 | chr19:0-4,704,133 | CN Loss | 4,704,134 | p13.3 | FSTL3, STK11, TCF3, GNA11, SH3GL1 |
| FTC07 | chr19:4,704,133-10,094,352 | CN Gain | 5,390,220 | p13.3 - p13.2 | MLLT1 |
| FTC07 | chr19:11,298,765-14,306,857 | CN Loss | 3,008,093 | p13.2 - p13.12 | LYL1 |
| FTC07 | chr19:14,306,857-18,252,711 | CN Gain | 3,945,855 | p13.12 - p13.11 | BRD4, TPM4, JAK3 |
| FTC08 | chr12:51,213,845-133,851,895 | CN Gain | 82,638,051 | q13.12 - q24.33 | ATF1, HOXC13, HOXC11, NACA, DDIT3, CDK4, LRIG3, WIF1, HMGA2, MDM2, BTG1, ALDH2, PTPN11, BCL7A |
| FTC08 | chr22:16,486,086-51,304,566 | CN Loss | 34,818,481 | q11.1 - q13.33 | CLTCL1, BCR, SMARCB1, MN1, CHEK2, EWSR1, NF2, MYH9, PDGFB, MKL1, EP300 |
| FTC10 | chr21:34,754,688-34,977,857 | CN Gain | 223,170 | q22.11 |  |
| FTC10 | chr22:16,486,086-51,304,566 | CN Loss | 34,818,481 | q11.1 - q13.33 | CLTCL1, BCR, SMARCB1, MN1, CHEK2, EWSR1, NF2, MYH9, PDGFB, MKL1, EP300 |
| FTC12 | chr1:17,341,060-18,459,888 | CN Gain | 1,118,829 | p36.13 | SDHB |
| FTC12 | chr1:37,952,294-116,706,006 | CN Loss | 78,753,713 | p34.3 - p13.1 | MYCL1, MPL, MUTYH, TAL1, CDKN2C, EPS15, JUN, JAK1, FUBP1, BCL10, RBM15, TRIM33, NRAS |
| FTC12 | chr1:156,700,514-249,250,621 | CN Loss | 92,550,108 | q23.1 - q44 | PRCC, NTRK1, SDHC, FCGR2B, PBX1, ABL2, TPR, MDM4, ELK4, SLC45A3, H3F3A, FH |
| FTC12 | chr2:0-90,265,119 | CN Loss | 90,265,120 | p25.3 - p11.2 | MYCN, C2orf44, NCOA1, DNMT3A, ALK, EML4, MSH2, MSH6, FBXO11, BCL11A, REL, XPO1 |
| FTC12 | chr2:95,529,039-243,199,373 | CN Loss | 147,670,335 | q11.1 - q37.3 | TTL, PAX8, ERCC3, CHN1, HOXD13, HOXD11, NFE2L2, PMS1, SF3B1, CREB1, IDH1, ATIC, FEV, PAX3, ACSL3 |
| FTC12 | chr6:37,259,712-38,332,083 | CN Gain | 1,072,372 | p21.2 |  |
| FTC12 | chr6:43,197,315-44,258,603 | CN Gain | 1,061,289 | p21.1 |  |
| FTC12 | chr7:0-57,558,200 | CN Gain | 57,558,201 | p22.3 - p11.2 | CARD11, PMS2, ETV1, HNRNPA2B1, HOXA9, HOXA11, HOXA13, JAZF1, IKZF1, EGFR |
| FTC12 | chr7:62,460,665-159,138,663 | CN Gain | 96,677,999 | q11.21 - q36.3 | SBDS, ELN, HIP1, AKAP9, CDK6, MET, SMO, CREB3L2, KIAA1549, BRAF, EZH2, MLL3 |
| FTC12 | chr9:82,144,032-141,213,431 | CN Loss | 59,069,400 | q21.31 - q34.3 | SYK, OMD, FANCC, XPA, NR4A3, TAL2, SET, FNBP1, ABL1, NUP214, TSC1, RALGDS, BRD3, NOTCH1 |
| FTC12 | chr21:14,678,356-16,552,201 | CN Loss | 1,873,846 | q11.2 - q21.1 |  |
| FTC13 | chr1:145,166,867-249,250,621 | CN Gain | 104,083,755 | q21.1 - q44 | BCL9, ARNT, TPM3, MUC1, PRCC, NTRK1, SDHC, FCGR2B, PBX1, ABL2, TPR, MDM4, ELK4, SLC45A3, H3F3A, FH |
| FTC13 | chr10:0-38,210,984 | CN Loss | 38,210,985 | p15.3 - p11.1 | GATA3, MLLT10, KIF5B |
| FTC13 | chr17:0-22,032,763 | CN Loss | 22,032,764 | p13.3 - p11.2 | YWHAE, USP6, TP53, PER1, GAS7, MAP2K4 |

* Array-CGH data in one FTA (FTA10) failed to pass the data quality control, and WES data was used for the CNA analysis instead.

* * Cancer Gene Census (http://cancer.sanger.ac.uk/census)
