# Supplementary material for: Mutational burdens and evolutionary ages of thyroid follicular adenoma are comparable to those of follicular carcinoma
Source: Oncotarget. 2016 Sep 9;7(43):69638–48. doi: 10.18632/oncotarget.11922 (PMC5342504; doi:10.18632/oncotarget.11922)
Supplement: Supplementary file 4 [file oncotarget-07-69638-s004.docx]

**Table S5. Pathway and ontology analysis of mutations**

| Category | Term | Count | *P* value (FTA) | *P* value (FTC) | Genes |
| --- | --- | --- | --- | --- | --- |
| GOTERM_BP | MAPKKK cascade | 8 | 0.808 | **0.002** | MAP4K3, MAP3K5, BRAF, FPR1, PAK1, LRRK2, GRM1, IL31RA |
| GOTERM_BP | cell adhesion | 16 | 0.282 | **0.004** | FERMT1, NEDD9, CASK, CELSR2, CDHR5, EDIL3, SLAMF7, ITGAM, CDH6, NRCAM, LAMA4, AGGF1, ACAN, MUC16, CDH23, CDH11 |
| GOTERM_BP | biological adhesion | 16 | 0.283 | **0.004** | FERMT1, NEDD9, CASK, CELSR2, CDHR5, EDIL3, SLAMF7, ITGAM, CDH6, NRCAM, LAMA4, AGGF1, ACAN, MUC16, CDH23, CDH11 |
| GOTERM_BP | actin cytoskeleton organization | 8 | **0.015** | **0.007** | NRAS, HRAS, WASF1, NEDD9, TTN, FHOD1, ADD1, ARHGEF11 |
| GOTERM_BP | actin filament-based process | 8 | **0.006** | **0.010** | NRAS, HRAS, WASF1, NEDD9, TTN, FHOD1, ADD1, ARHGEF11 |
| GOTERM_BP | cytoskeleton organization | 11 | **0.040** | **0.011** | NRAS, HRAS, WASF1, DMD, NEDD9, PAK1, TTN, PCLO, FHOD1, ADD1, ARHGEF11 |
| GOTERM_BP | intracellular signaling cascade | 22 | 0.564 | **0.011** | HRAS, ADCY2, BRAF, CCKBR, FPR1, MED12, BRIP1, ARID1A, GRM1, PCLO, LATS1, ASB18, IL31RA, ARHGEF11, MAP4K3, NRAS, MAP3K5, NMUR2, CHN1, PAK1, LRRK2, MLL3 |
| GOTERM_BP | cell-cell adhesion | 8 | 0.445 | **0.019** | NRCAM, ACAN, CDHR5, CELSR2, ITGAM, CDH11, CDH6, CDH23 |
| GOTERM_BP | regulation of programmed cell death | 15 | 0.106 | **0.029** | MUC2, HRAS, BRAF, TP53BP2, BIRC6, SLAMF7, IL31RA, ARHGEF11, NRAS, SON, MAP3K5, RPS3A, TXNDC5, RYR2, LRRK2 |
| GOTERM_BP | regulation of cell death | 15 | 0.108 | **0.029** | MUC2, HRAS, BRAF, TP53BP2, BIRC6, SLAMF7, IL31RA, ARHGEF11, NRAS, SON, MAP3K5, RPS3A, TXNDC5, RYR2, LRRK2 |
| GOTERM_BP | protein amino acid phosphorylation | 13 | 0.240 | **0.031** | MAP4K3, MAP3K5, BRAF, FPR1, CASK, PAK1, LRRK2, TTN, FPGT, LATS1, PIK3R4, TSSK3, GRM1 |
| GOTERM_BP | actin filament organization | 4 | - | **0.034** | WASF1, NEDD9, TTN, ADD1 |
| GOTERM_BP | positive regulation of Rac protein signal transduction | 2 | **0.035** | **0.039** | NRAS, HRAS |
| GOTERM_BP | detection of chemical stimulus | 3 | - | **0.039** | KCNMB3, TRPA1, RYR2 |
| GOTERM_BP | homophilic cell adhesion | 5 | - | **0.040** | CDHR5, CELSR2, CDH11, CDH6, CDH23 |
| GOTERM_BP | dicarboxylic acid metabolic process | 3 | - | **0.044** | CS, ACMSD, LIPF |
| GOTERM_BP | transcytosis | 2 | - | **0.048** | TG, LRP2 |
| GOTERM_BP | regulation of apoptosis | 14 | 0.101 | **0.053** | MUC2, HRAS, BRAF, TP53BP2, BIRC6, SLAMF7, ARHGEF11, IL31RA, NRAS, SON, MAP3K5, RPS3A, TXNDC5, RYR2 |
| GOTERM_BP | regulation of protein kinase activity | 8 | 0.192 | **0.054** | ZGPAT, MAP3K5, ADCY2, FPR1, PAK1, TTN, GRM1, LATS1 |
| GOTERM_BP | regulation of kinase activity | 8 | 0.211 | **0.063** | ZGPAT, MAP3K5, ADCY2, FPR1, PAK1, TTN, GRM1, LATS1 |
| GOTERM_BP | protein kinase cascade | 8 | 0.642 | **0.073** | MAP4K3, MAP3K5, BRAF, FPR1, PAK1, LRRK2, GRM1, IL31RA |
| GOTERM_BP | regulation of transferase activity | 8 | 0.235 | **0.074** | ZGPAT, MAP3K5, ADCY2, FPR1, PAK1, TTN, GRM1, LATS1 |
| GOTERM_BP | striated muscle contraction | 3 | 0.337 | **0.075** | RYR2, TTN, ARHGEF11 |
| GOTERM_BP | positive regulation of MAP kinase activity | 4 | - | **0.079** | MAP3K5, FPR1, PAK1, GRM1 |
| GOTERM_BP | second-messenger-mediated signaling | 6 | 0.879 | **0.082** | ADCY2, CCKBR, NMUR2, FPR1, PCLO, GRM1 |
| GOTERM_BP | detection of calcium ion | 2 | - | **0.085** | KCNMB3, RYR2 |
| GOTERM_BP | regulation of phosphorylation | 9 | 0.120 | **0.089** | ZGPAT, MAP3K5, ADCY2, FPR1, PAK1, TTN, GRM1, LATS1, IL31RA |
| GOTERM_BP | behavior | 9 | 0.237 | **0.091** | NRAS, HRAS, CCKBR, NMUR2, FPR1, STRN, GRM1, ITGAM, CDH23 |
| GOTERM_BP | regulation of MAPKKK cascade | 4 | 0.252 | **0.092** | MAP3K5, ATP6AP2, PAK1, GRM1 |
| GOTERM_BP | axonal fasciculation | 2 | - | **0.094** | NRCAM, NDN |
| GOTERM_BP | blood vessel development | 6 | 0.643 | **0.094** | ARHGAP22, LAMA4, AGGF1, DHCR7, MMP19, CSPG4 |
| GOTERM_BP | phosphorylation | 13 | 0.416 | **0.095** | MAP4K3, MAP3K5, BRAF, FPR1, CASK, PAK1, LRRK2, TTN, FPGT, LATS1, PIK3R4, TSSK3, GRM1 |
| GOTERM_BP | induction of apoptosis | 8 | **0.024** | **0.096** | MUC2, NOTCH2, PRUNE2, NR4A1, RYR2, TP63, FEM1B, CUL1 |
| GOTERM_BP | cellular component morphogenesis | 8 | 0.871 | **0.097** | NRCAM, NDN, DMD, CELSR2, TTN, ADD1, ARHGEF11, CDH23 |
| GOTERM_BP | induction of programmed cell death | 8 | **0.024** | **0.097** | MUC2, NOTCH2, PRUNE2, NR4A1, RYR2, TP63, FEM1B, CUL1 |
| GOTERM_BP | positive regulation of programmed cell death | 9 | **0.039** | 0.135 | MUC2, NOTCH2, PRUNE2, NF1, NR4A1, RYR2, TP63, FEM1B, CUL1 |
| GOTERM_BP | neurological system process | 17 | **0.069** | 0.137 | HRAS, MYO1A, OR5P2, OR5M11, POU6F2, NF1, NRAS, OR4C13, NAALAD2, EYS, BEST1, CRB1, OR13D1, OR5AP2, OR10R2, CACNA1C, USH2A |
| GOTERM_BP | positive regulation of cell death | 9 | **0.040** | 0.137 | MUC2, NOTCH2, PRUNE2, NF1, NR4A1, RYR2, TP63, FEM1B, CUL1 |
| GOTERM_BP | positive regulation of Ras protein signal transduction | 3 | **0.009** | 0.146 | NRAS, NOTCH2, HRAS |
| GOTERM_BP | positive regulation of small GTPase mediated signal transduction | 3 | **0.010** | 0.155 | NRAS, NOTCH2, HRAS |
| GOTERM_BP | muscle contraction | 6 | **0.012** | 0.191 | MYH4, RYR2, MYH13, TTN, CACNA1C, GJA5 |
| GOTERM_BP | muscle system process | 6 | **0.017** | 0.229 | MYH4, RYR2, MYH13, TTN, CACNA1C, GJA5 |
| GOTERM_BP | visual learning | 4 | **0.002** | 0.234 | NRAS, HRAS, NF1, CACNA1C |
| GOTERM_BP | regulation of cell proliferation | 14 | **0.022** | 0.246 | MUC2, NOTCH2, NRAS, HRAS, NOP2, NF2, TBC1D8, TAF6, AGGF1, NF1, PDGFRB, TP63, TSHR, CUL1 |
| GOTERM_BP | positive regulation of apoptosis | 9 | **0.038** | 0.249 | MUC2, NOTCH2, PRUNE2, NF1, NR4A1, RYR2, TP63, FEM1B, CUL1 |
| GOTERM_BP | visual behavior | 4 | **0.003** | 0.264 | NRAS, HRAS, NF1, CACNA1C |
| GOTERM_BP | cell aging | 3 | **0.034** | 0.279 | NRAS, HRAS, TP63 |
| GOTERM_BP | muscle cell differentiation | 4 | **0.093** | 0.334 | NRAS, HRAS, SYNE1, TTN |
| GOTERM_BP | regulation of Ras protein signal transduction | 7 | **0.011** | 0.341 | AGAP5, NRAS, NOTCH2, HRAS, TBC1D8, NF1, TTN |
| GOTERM_BP | muscle organ development | 6 | **0.040** | 0.344 | COL19A1, NF1, COL6A3, PDGFRB, TP63, TTN |
| GOTERM_BP | positive regulation of cell proliferation | 8 | **0.075** | 0.385 | NRAS, HRAS, NOP2, TBC1D8, AGGF1, PDGFRB, TP63, TSHR |
| GOTERM_BP | Notch signaling pathway | 3 | **0.078** | 0.402 | NOTCH2, TP63, MAML3 |
| GOTERM_BP | cognition | 16 | **0.014** | 0.404 | HRAS, MYO1A, OR5P2, OR5M11, POU6F2, NF1, NRAS, OR4C13, EYS, BEST1, CRB1, OR13D1, OR5AP2, OR10R2, CACNA1C, USH2A |
| GOTERM_BP | learning | 4 | **0.016** | 0.448 | NRAS, HRAS, NF1, CACNA1C |
| GOTERM_BP | regulation of small GTPase mediated signal transduction | 7 | **0.025** | 0.452 | AGAP5, NRAS, NOTCH2, HRAS, TBC1D8, NF1, TTN |
| GOTERM_BP | multicellular organismal homeostasis | 4 | **0.040** | 0.569 | MUC2, SLC11A1, PDGFRB, MUC6 |
| GOTERM_BP | learning or memory | 4 | **0.076** | 0.668 | NRAS, HRAS, NF1, CACNA1C |
| GOTERM_BP | chromosome organization | 9 | **0.067** | 0.707 | EHMT1, NIPBL, EZH1, TAF6L, BCOR, TTN, MLL3, KDM5C, CENPH |
| GOTERM_BP | muscle tissue development | 5 | **0.025** | 0.711 | COL19A1, NF1, PDGFRB, TP63, TTN |
| GOTERM_BP | regulation of locomotion | 5 | **0.091** | 0.852 | MUC2, NF2, NF1, PDGFRB, TSHR |
| GOTERM_BP | chromatin modification | 6 | **0.097** | 0.935 | EHMT1, EZH1, TAF6L, BCOR, MLL3, KDM5C |
| GOTERM_BP | smooth muscle tissue development | 3 | **0.003** | - | NF1, PDGFRB, TP63 |
| GOTERM_BP | collagen fibril organization | 3 | **0.027** | - | COL14A1, LMX1B, NF1 |
| GOTERM_BP | sympathetic nervous system development | 2 | **0.044** | - | NF1, TP63 |
| GOTERM_BP | epithelial structure maintenance | 2 | **0.052** | - | MUC2, MUC6 |
| GOTERM_BP | maintenance of gastrointestinal epithelium | 2 | **0.052** | - | MUC2, MUC6 |
| GOTERM_BP | extracellular matrix organization | 4 | **0.065** | - | COL14A1, COL19A1, LMX1B, NF1 |
| GOTERM_BP | odontogenesis | 3 | **0.083** | - | NF2, TP63, BCOR |
| GOTERM_BP | negative regulation of cell migration | 3 | **0.091** | - | MUC2, NF2, NF1 |
| GOTERM_CC | cytoskeleton | 27 | **0.046** | **0.001** | ENAH, NDN, WASF1, FERMT1, STRN, CASK, NEDD9, TTN, LATS1, KRTAP13-1, MAP1LC3B, DMD, KRT85, STAG2, CDH23, MED12, KRTAP9-8, MCM3, CDC27, PCLO, GRM1, MYH8, MID2, KRTAP4-8, ZNF98, FHOD1, ADD1 |
| GOTERM_CC | cytoskeletal part | 20 | **0.027** | **0.003** | NDN, FERMT1, MED12, NEDD9, STRN, TTN, MCM3, KRTAP9-8, CDC27, LATS1, GRM1, MYH8, MID2, KRTAP13-1, MAP1LC3B, KRTAP4-8, KRT85, ZNF98, ADD1, CDH23 |
| GOTERM_CC | synapse | 10 | 0.635 | **0.010** | ENAH, CTBP2, GABRB1, DMD, CASK, STRN, LRRK2, PCLO, GRM1, CDH23 |
| GOTERM_CC | non-membrane-bounded organelle | 38 | 0.337 | **0.016** | ENAH, NDN, FOXK1, WASF1, FERMT1, STRN, NEDD9, CASK, TTN, LATS1, KRTAP13-1, RRN3, DGCR8, H2AFV, RPS3A, MAP1LC3B, DMD, KRT85, STAG2, CDH23, TRPA1, MED12, ARID1A, KRTAP9-8, MCM3, CDC27, MYH8, PCLO, GRM1, ARHGEF11, MID2, KRTAP4-8, RBM19, MYBBP1A, ZNF98, FPGT, FHOD1, ADD1 |
| GOTERM_CC | intracellular non-membrane-bounded organelle | 38 | 0.337 | **0.016** | ENAH, NDN, FOXK1, WASF1, FERMT1, STRN, NEDD9, CASK, TTN, LATS1, KRTAP13-1, RRN3, DGCR8, H2AFV, RPS3A, MAP1LC3B, DMD, KRT85, STAG2, CDH23, TRPA1, MED12, ARID1A, KRTAP9-8, MCM3, CDC27, MYH8, PCLO, GRM1, ARHGEF11, MID2, KRTAP4-8, RBM19, MYBBP1A, ZNF98, FPGT, FHOD1, ADD1 |
| GOTERM_CC | basolateral plasma membrane | 7 | 0.557 | **0.018** | ENAH, DMD, MUC20, FERMT1, CASK, NEDD9, PAK1 |
| GOTERM_CC | cell projection | 14 | 0.106 | **0.025** | ENAH, ADCY2, WASF1, MUC20, CSPG4, FERMT1, NEDD9, STRN, GRM1, NRCAM, OXCT2, LRRK2, LRP2, CDH23 |
| GOTERM_CC | cell-substrate junction | 5 | - | **0.027** | ENAH, DMD, FERMT1, NEDD9, PAK1 |
| GOTERM_CC | plasma membrane | 49 | 0.343 | **0.042** | HRAS, ENAH, ADCY2, ATP6AP2, GABRB1, OR10T2, CSPG4, FERMT1, CASK, STRN, IL31RA, NRCAM, NMUR2, PAK1, FLVCR2, CDH23, CTBP2, CCKBR, BRAF, ABCB11, TRPA1, OR10J5, CDHR5, OR5M9, GRM1, PCLO, ARHGEF11, LILRB1, RYR2, LRRK2, ADD1, KCNMB3, OR5D13, FPR1, NEDD9, ITGAM, CDH6, DMD, POTEC, MUC20, ATP1A4, CELSR2, NRAS, DYTN, LRP2, ABCC8, FPGT, MUC16, CDH11 |
| GOTERM_CC | keratin filament | 4 | **0.047** | **0.060** | KRTAP4-8, KRTAP9-8, KRT85, ZNF98 |
| GOTERM_CC | membrane fraction | 14 | 0.206 | **0.068** | HRAS, CCKBR, ABCB11, BIRC6, CASK, GRM1, NRAS, PSG9, FMO1, DMD, CYP4Z1, RYR2, LRRK2, LRP2 |
| GOTERM_CC | Z disc | 3 | **0.066** | **0.080** | DMD, RYR2, TTN |
| GOTERM_CC | cell junction | 10 | 0.859 | **0.082** | ENAH, CTBP2, ABCB11, GABRB1, DMD, FERMT1, STRN, NEDD9, PAK1, PCLO |
| GOTERM_CC | insoluble fraction | 14 | 0.238 | **0.085** | HRAS, CCKBR, ABCB11, BIRC6, CASK, GRM1, NRAS, PSG9, FMO1, DMD, CYP4Z1, RYR2, LRRK2, LRP2 |
| GOTERM_CC | focal adhesion | 4 | - | **0.086** | ENAH, FERMT1, NEDD9, PAK1 |
| GOTERM_CC | cell-substrate adherens junction | 4 | - | **0.094** | ENAH, FERMT1, NEDD9, PAK1 |
| GOTERM_CC | myofibril | 7 | **0.001** | 0.104 | MYO18B, SYNE1, MYH4, RYR2, MYH13, TTN, CACNA1C |
| GOTERM_CC | I band | 3 | **0.090** | 0.108 | RYR2, TTN, CACNA1C |
| GOTERM_CC | contractile fiber part | 7 | **0.001** | 0.108 | MYO18B, SYNE1, MYH4, RYR2, MYH13, TTN, CACNA1C |
| GOTERM_CC | intermediate filament | 6 | **0.026** | 0.116 | KRT9, KRTAP4-9, KRTAP16-1, KRT7, KRT13, KRTAP6-3 |
| GOTERM_CC | intermediate filament cytoskeleton | 6 | **0.029** | 0.123 | KRT9, KRTAP4-9, KRTAP16-1, KRT7, KRT13, KRTAP6-3 |
| GOTERM_CC | contractile fiber | 7 | **0.001** | 0.126 | MYO18B, SYNE1, MYH4, RYR2, MYH13, TTN, CACNA1C |
| GOTERM_CC | sarcomere | 6 | **0.002** | 0.263 | MYO18B, SYNE1, MYH4, RYR2, TTN, CACNA1C |
| GOTERM_CC | microvillus | 3 | **0.043** | 0.308 | MYO1A, MSN, DOCK4 |
| GOTERM_CC | extracellular matrix part | 5 | **0.022** | 0.334 | COL14A1, COL19A1, COL6A3, GRIA3, USH2A |
| GOTERM_CC | proteinaceous extracellular matrix | 7 | **0.074** | 0.411 | MUC2, COL14A1, COL19A1, COL6A3, THSD4, PRSS36, USH2A |
| GOTERM_CC | extracellular matrix | 8 | **0.039** | 0.467 | MUC2, COL14A1, COL19A1, COL6A3, THSD4, GRIA3, PRSS36, USH2A |
| GOTERM_CC | myosin complex | 4 | **0.022** | - | MYO18B, MYO1A, MYH4, MYH13 |
| GOTERM_CC | collagen | 3 | **0.041** | - | COL14A1, COL19A1, COL6A3 |
| GOTERM_CC | anchoring collagen | 2 | **0.079** | - | COL14A1, COL6A3 |
| GOTERM_MF | protein serine/threonine kinase activity | 11 | 0.801 | **0.010** | MAP4K3, MAP3K5, BRAF, CASK, PAK1, TTN, LRRK2, TSSK3, PIK3R4, FPGT, LATS1 |
| GOTERM_MF | calmodulin binding | 6 | **0.012** | **0.012** | CASK, STRN, RYR2, TTN, MYH8, ADD1 |
| GOTERM_MF | nucleotide binding | 33 | 0.106 | **0.015** | ABCF3, HRAS, ADCY2, ABCD1, CASK, IGF2BP3, TTN, LATS1, DDX27, MAP3K5, OPLAH, FMO1, NMUR2, PAK1, PIK3R4, CTBP2, BRAF, ABCB11, SACS, BRIP1, ATP1A4, MCM3, CNGA4, MYH8, HNRNPR, MAP4K3, NRAS, RBM19, LRRK2, ABCC8, FPGT, TSSK3, REV3L |
| GOTERM_MF | calcium ion binding | 17 | 0.558 | **0.017** | SRL, MMP19, CELSR2, CDHR5, EDIL3, TTN, PCLO, ITGAM, CDH6, DYTN, DMD, GALNS, RYR2, LRP2, CDH23, COL10A1, CDH11 |
| GOTERM_MF | adenyl nucleotide binding | 25 | **0.034** | **0.018** | ABCF3, ADCY2, BRAF, ABCB11, ABCD1, SACS, BRIP1, ATP1A4, CASK, MCM3, TTN, CNGA4, MYH8, LATS1, MAP4K3, DDX27, MAP3K5, OPLAH, FMO1, PAK1, LRRK2, ABCC8, TSSK3, PIK3R4, FPGT |
| GOTERM_MF | adenyl ribonucleotide binding | 24 | **0.020** | **0.018** | ABCF3, ADCY2, BRAF, ABCB11, ABCD1, SACS, BRIP1, ATP1A4, CASK, TTN, MCM3, CNGA4, LATS1, MYH8, MAP4K3, DDX27, MAP3K5, OPLAH, PAK1, LRRK2, ABCC8, FPGT, TSSK3, PIK3R4 |
| GOTERM_MF | purine nucleoside binding | 25 | **0.040** | **0.021** | ABCF3, ADCY2, BRAF, ABCB11, ABCD1, SACS, BRIP1, ATP1A4, CASK, MCM3, TTN, CNGA4, MYH8, LATS1, MAP4K3, DDX27, MAP3K5, OPLAH, FMO1, PAK1, LRRK2, ABCC8, TSSK3, PIK3R4, FPGT |
| GOTERM_MF | nucleoside binding | 25 | **0.043** | **0.023** | ABCF3, ADCY2, BRAF, ABCB11, ABCD1, SACS, BRIP1, ATP1A4, CASK, MCM3, TTN, CNGA4, MYH8, LATS1, MAP4K3, DDX27, MAP3K5, OPLAH, FMO1, PAK1, LRRK2, ABCC8, TSSK3, PIK3R4, FPGT |
| GOTERM_MF | ATP binding | 24 | **0.017** | **0.029** | KIFC2, MYO1A, CARS, NLRP5, NIN, STRADA, MYH4, OAS1, TTN, DNAH7, NLRP2, DDX58, STYK1, MYO18B, KIF7, CSNK1D, LACE1, MAP3K9, PDGFRB, ETNK1, MYH13, JAK3, DDX53, DCLK1 |
| GOTERM_MF | purine nucleotide binding | 28 | **0.054** | **0.030** | HRAS, ABCF3, ADCY2, ABCD1, CASK, TTN, LATS1, DDX27, MAP3K5, OPLAH, FMO1, NMUR2, PAK1, PIK3R4, BRAF, ABCB11, SACS, ATP1A4, BRIP1, MCM3, CNGA4, MYH8, MAP4K3, NRAS, LRRK2, ABCC8, FPGT, TSSK3 |
| GOTERM_MF | ribonucleotide binding | 27 | **0.034** | **0.031** | HRAS, ABCF3, ADCY2, ABCD1, CASK, TTN, LATS1, DDX27, MAP3K5, OPLAH, NMUR2, PAK1, PIK3R4, BRAF, ABCB11, SACS, ATP1A4, BRIP1, MCM3, CNGA4, MYH8, MAP4K3, NRAS, LRRK2, FPGT, TSSK3, ABCC8 |
| GOTERM_MF | purine ribonucleotide binding | 27 | **0.034** | **0.031** | HRAS, ABCF3, ADCY2, ABCD1, CASK, TTN, LATS1, DDX27, MAP3K5, OPLAH, NMUR2, PAK1, PIK3R4, BRAF, ABCB11, SACS, ATP1A4, BRIP1, MCM3, CNGA4, MYH8, MAP4K3, NRAS, LRRK2, FPGT, TSSK3, ABCC8 |
| GOTERM_MF | metal ion binding | 51 | 0.887 | **0.044** | ADCY2, ZNF81, DZIP1, EDIL3, TTN, LATS1, ZGPAT, MAP3K5, CH25H, MLL3, ZNF180, CDH23, COL10A1, ZCCHC8, BRAF, MMP19, CDHR5, PCLO, ZDHHC11, RYR2, CHSY1, ZNF746, ZNF98, ADD1, REV3L, SRL, ITGAM, TRIML1, CDH6, ARIH1, TCF20, DMD, GALNS, ZNF425, DTX4, MGAT4B, AGBL1, ATP1A4, CELSR2, MID2, DYTN, PHF14, CYP4Z1, CHN1, HIVEP3, ADAM21, ZBTB3, LRP2, FPGT, TSSK3, CDH11 |
| GOTERM_MF | ion binding | 52 | 0.843 | **0.045** | ADCY2, ZNF81, GABRB1, DZIP1, EDIL3, TTN, LATS1, ZGPAT, MAP3K5, CH25H, MLL3, ZNF180, CDH23, COL10A1, ZCCHC8, BRAF, MMP19, CDHR5, PCLO, ZDHHC11, RYR2, CHSY1, ZNF746, ZNF98, ADD1, REV3L, SRL, ITGAM, TRIML1, CDH6, ARIH1, TCF20, DMD, GALNS, ZNF425, DTX4, MGAT4B, AGBL1, ATP1A4, CELSR2, MID2, DYTN, PHF14, CYP4Z1, HIVEP3, CHN1, ADAM21, ZBTB3, LRP2, FPGT, TSSK3, CDH11 |
| GOTERM_MF | cation binding | 51 | 0.861 | **0.052** | ADCY2, ZNF81, DZIP1, EDIL3, TTN, LATS1, ZGPAT, MAP3K5, CH25H, MLL3, ZNF180, CDH23, COL10A1, ZCCHC8, BRAF, MMP19, CDHR5, PCLO, ZDHHC11, RYR2, CHSY1, ZNF746, ZNF98, ADD1, REV3L, SRL, ITGAM, TRIML1, CDH6, ARIH1, TCF20, DMD, GALNS, ZNF425, DTX4, MGAT4B, AGBL1, ATP1A4, CELSR2, MID2, DYTN, PHF14, CYP4Z1, CHN1, HIVEP3, ADAM21, ZBTB3, LRP2, FPGT, TSSK3, CDH11 |
| GOTERM_MF | cytoskeletal protein binding | 11 | **0.027** | **0.059** | PHACTR1, FMNL3, MYO18B, SYNE1, MYO1A, NF2, LIMCH1, MYH4, MYH13, MSN, TTN |
| GOTERM_MF | structural constituent of muscle | 3 | 0.341 | **0.063** | DMD, TTN, MYH8 |
| GOTERM_MF | protein kinase activity | 11 | 0.248 | **0.073** | MAP4K3, MAP3K5, BRAF, CASK, PAK1, TTN, LRRK2, TSSK3, PIK3R4, FPGT, LATS1 |
| GOTERM_MF | ATPase activity, coupled to transmembrane movement of substances | 4 | - | **0.091** | ABCB11, ABCD1, ATP1A4, ABCC8 |
| GOTERM_MF | ATPase activity, coupled to movement of substances | 4 | - | **0.093** | ABCB11, ABCD1, ATP1A4, ABCC8 |
| GOTERM_MF | hydrolase activity, acting on acid anhydrides, catalyzing transmembrane movement of substances | 4 | - | **0.095** | ABCB11, ABCD1, ATP1A4, ABCC8 |
| GOTERM_MF | actin binding | 10 | **0.005** | 0.101 | PHACTR1, FMNL3, MYO18B, SYNE1, MYO1A, LIMCH1, MYH4, MYH13, MSN, TTN |
| GOTERM_MF | extracellular matrix structural constituent | 4 | **0.053** | 0.206 | MUC2, COL14A1, COL19A1, MUC6 |
| GOTERM_MF | protein tyrosine kinase activity | 5 | **0.082** | 0.485 | STYK1, NIN, PDGFRB, JAK3, TTN |
| GOTERM_MF | motor activity | 7 | **0.003** | - | KIFC2, MYO18B, MYO1A, KIF7, MYH4, MYH13, DNAH7 |
| GOTERM_MF | protein methyltransferase activity | 4 | **0.013** | - | EHMT1, EZH1, PCMTD1, MLL3 |
| GOTERM_MF | lysine N-methyltransferase activity | 3 | **0.039** | - | EHMT1, EZH1, MLL3 |
| GOTERM_MF | protein-lysine N-methyltransferase activity | 3 | **0.039** | - | EHMT1, EZH1, MLL3 |
| GOTERM_MF | histone-lysine N-methyltransferase activity | 3 | **0.039** | - | EHMT1, EZH1, MLL3 |
| GOTERM_MF | histone methyltransferase activity | 3 | **0.056** | - | EHMT1, EZH1, MLL3 |
| GOTERM_MF | N-methyltransferase activity | 3 | **0.096** | - | EHMT1, EZH1, MLL3 |
| BIOCARTA | MAPKinase Signaling Pathway | 5 | 0.469 | **0.026** | MAP4K3, HRAS, MAP3K5, BRAF, PAK1 |
| BIOCARTA | Synaptic Proteins at the Synaptic Junction | 3 | - | **0.027** | CASK, PCLO, ADD1 |
| BIOCARTA | Rac 1 cell motility signaling pathway | 3 | - | **0.033** | WASF1, CHN1, PAK1 |
| BIOCARTA | fMLP induced chemokine gene expression in HMC-1 cells | 3 | - | **0.050** | HRAS, FPR1, PAK1 |
| KEGG | Regulation of actin cytoskeleton | 7 | 0.143 | **0.046** | NRAS, HRAS, ENAH, BRAF, WASF1, PAK1, ITGAM |
| KEGG | Thyroid cancer | 3 | 0.241 | **0.048** | NRAS, HRAS, BRAF |
| KEGG | Long-term potentiation | 4 | 0.134 | **0.049** | NRAS, HRAS, BRAF, GRM1 |
| KEGG | Long-term depression | 4 | 0.138 | **0.051** | NRAS, HRAS, BRAF, GRM1 |
| KEGG | Renal cell carcinoma | 4 | 0.488 | **0.053** | NRAS, HRAS, BRAF, PAK1 |
| KEGG | Chronic myeloid leukemia | 4 | 0.512 | **0.063** | NRAS, HRAS, CTBP2, BRAF |
| KEGG | ErbB signaling pathway | 4 | 0.565 | **0.089** | NRAS, HRAS, BRAF, PAK1 |
| KEGG | Bladder cancer | 3 | 0.330 | **0.092** | NRAS, HRAS, BRAF |
| KEGG | Gap junction | 4 | **0.051** | **0.094** | NRAS, HRAS, ADCY2, GRM1 |
| PANTHER | Inflammation mediated by chemokine and cytokine signaling pathway | 10 | **0.002** | **0.007** | NRAS, HRAS, ADCY2, BRAF, FPR1, CASK, PAK1, TTN, MYH8, ITGAM |
| PANTHER | Integrin signalling pathway | 8 | **0.058** | **0.013** | NRAS, HRAS, ENAH, LAMA4, MAP3K5, BRAF, ITGAM, COL10A1 |
| PANTHER | Cytoskeletal regulation by Rho GTPase | 5 | 0.290 | **0.039** | ENAH, PAK1, TTN, MYH8, ARHGEF11 |
| PANTHER | Ras Pathway | 4 | 0.594 | **0.090** | NRAS, HRAS, BRAF, PAK1 |
| PANTHER | Wnt signaling pathway | 8 | 0.854 | **0.091** | CTBP2, CDHR5, ARID1A, CELSR2, MYH8, CDH11, CDH6, CDH23 |
| PANTHER | Nicotinic acetylcholine receptor signaling pathway | 6 | **0.001** | - | MYO18B, CTAGE5, MYO1A, MYH4, MYH13, CACNA1C |
